# Disrupted hierarchical functional brain organization in affective and psychotic disorders: insights from functional brain gradients

**DOI:** 10.1038/s41398-026-04206-z

**Published:** 2026-07-29

**Authors:** Hannah Hacker, Linnea Hoheisel, Madalina-Octavia Buciuman, Annkathrin Böke, Theresa Lichtenstein, Marlene Rosen, Shalaila S. Haas, Anne Ruef, Dominic B. Dwyer, Paolo Brambilla, Carolina Bonivento, Rachel Upthegrove, Stephen J. Wood, Stefan Borgwardt, Eva Meisenzahl, Stephan Ruhrmann, Raimo K. R. Salokangas, Alessandro Bertolino, Rebekka Lencer, Udo Dannlowski, Nikolaos Koutsouleris, Lana Kambeitz-Ilankovic, Joseph Kambeitz

**Affiliations:** 1 Department of Psychiatry and Psychotherapy, Faculty of Medicine and University Hospital of Cologne, University of Cologne, Cologne, Germany; 2 Institute of Neuroscience and Medicine (INM-3), Forschungszentrum Jülich, Jülich, Germany; 3 Department of Psychiatry and Psychotherapy, Ludwig-Maximilian University, München, Germany; 4 Department of Psychiatry, Icahn School of Medicine at Mount Sinai, New York, NY USA; 5 Orygen, Melbourne, Australia; 6 Department of Neurosciences and Mental Health, IRCCS Ca’ Granda Ospedale Maggiore Policlinico, Milan, Italy; 7 Department of Pathophysiology and Transplantation, University of Milan, Milano, Italy; 8 Scientific Institute, IRCCS E. Medea, Pasian di Prato, Udine, Italy; 9 Department of Psychiatry, University of Oxford, Oxford, UK; 10 Institute for Mental Health, University of Birmingham, Birmingham, UK; 11 Centre for Mental Health and Brain Sciences, Swinburne University of Technology, Hawthorn, VIC Australia; 12 Centre for Youth Mental Health, University of Melbourne, Parkville, Australia; 13 Department of Psychiatry and Psychotherapy, University of Luebeck, Lübeck, Germany; 14 Department of Psychiatry and Psychotherapy, Medical Faculty, Heinrich-Heine-University, Düsseldorf, Germany; 15 Department of Psychiatry, University of Turku, Turku, Finland; 16 Department of Translational Biomedicine and Neuroscience University of Bari Aldo Moro, Bari, Italy; 17 Institute for Translational Psychiatry, University of Münster, Münster, Germany; 18 Department of Psychiatry, Medical School and University Medical Center OWL, Protestant Hospital of the Bethel Foundation, Bielefeld University, Bielefeld, Germany; 19 German Center for Mental Health (DZPG), Site Jena Magdeburg Halle, Jena, Germany; 20 Center for Intervention and Research on Adaptive and Maladaptive Brain Circuits Underlying Mental Health (C-I-R-C), Site Jena Magdeburg Halle, Jena, Germany; 21 Department of Psychosis Studies, Institute of Psychiatry, Psychology and Neuroscience, King´s College London, London, UK; 22 Max Planck Institute for Psychiatry, München, Germany; 23 Faculty of Psychology and Educational Sciences, Department of Psychology, Ludwig-Maximilian University, München, Germany

**Keywords:** Depression, Schizophrenia

## Abstract

Individuals with psychosis and depression show widespread alterations in brain resting-state functional connectivity (rs-FC), affecting both sensory and higher-order brain regions. In this study, we investigate disruptions in the hierarchical organization of brain functional networks in individuals with psychotic and affective disorders. We derived functional brain gradients, low dimensional representations of rs-FC that capture cortical hierarchy, in a sample of 1071 (56.3% female) participants, including clinical high-risk for psychosis (CHR-P) individuals, recent-onset psychosis (ROP) patients, recent-onset depression (ROD) patients, and healthy controls (HC). We examined regional alterations, network-level alterations and functional differentiation and their relationship to clinical symptoms. In addition, we linked case-control differences to receptor expression maps to explore underlying neurobiological mechanisms. All clinical groups exhibited alterations in the visual-to-sensorimotor gradient, while only ROP patients showed alterations in the sensory-to-association gradient. CHR-P and ROP individuals exhibited lower values in the ventral attention network. Clinical groups combined showed higher values in the somatomotor network, a reduced gradient range and altered between-network dispersion. ROD patients showed reduced within-network dispersion in the attentional networks and a reduced range. Correlational analysis revealed weak associations of gradient measures with functioning, visual dysfunctions and cognition. Case-control differences showed associations to receptor expression maps, suggesting the involvement of neurotransmitter systems in these disruptions. Our findings reveal transdiagnostic and disease-specific alterations of hierarchical brain organization. These alterations indicate deficits in functional integration across psychiatric diseases, highlighting the role of attentional and sensory networks in disease processes.

## Introduction

Psychotic and affective disorders represent a major burden for individuals and society due to their high prevalence and impact on psychosocial functioning [1]. Affected individuals experience a wide range of symptoms, including those in affective, attentional and sensory domains. Despite the distinct symptoms associated with each disorder, affective and psychotic syndromes frequently co-occur [2]. About 20% of individuals with depression experience psychotic symptoms [3] and 40% of individuals with schizophrenia also experience a depression [4]. Beyond shared clinical symptoms, increasing evidence suggests overlapping neurobiological abnormalities [5–8], including altered brain functional connectivity (FC) [9] and neurotransmitter systems [10]. Overlapping neurostructural subtypes have also been identified for psychosis and depression [5]. These overlaps raise the question whether these diseases share underlying neural alterations and underline the need to identify shared disease mechanisms.

Previous research has shown that resting-state functional connectivity (rs-FC) is altered in patients with depression, schizophrenia and even in individuals with clinical high-risk for psychosis (CHR-P) [11–13]. Observed alterations are present in multiple brain networks [13–18] including sensory processing areas and areas relevant to higher-order processes such as the control network (CN), the default mode network (DMN) and the dorsal attention network (DAN) [15, 16].

While some studies point to regional alterations of rs-FC in psychosis and depression, there is increasing evidence indicating changes in overall *network structure* [19, 20].

This observation has motivated novel analysis approaches focusing on the *hierarchical* organization of brain rs-FC in these disorders, aiming to capture large-scale patterns. One approach detects these patterns by mapping brain regions along continuous axes, referred to as *functional brain gradients* [21]. Through a low-dimensional representation of rs-FC, these gradients capture how similar regions are in their patterns of coactivation, providing insights of how functional areas are organized across the cortex [22]. This approach might provide novel insights regarding the neurobiological basis for symptoms observed in depression and psychosis. Typically, the first gradient extends from unimodal sensory areas to trans-modal association areas like the DMN (sensory-to-association gradient), while the second gradient extends from visual to sensorimotor areas (visual-to-sensorimotor gradient) [21]. However, the ordering of the gradients is influenced by the amount of variance explained during dimensionality reduction and therefore it is possible that the gradient order does not necessarily reflect these two axes in this order [23, 24]. These gradients have been found to correspond to myelination, macrostructural features [21], cortical thickness and receptor expression [25]. It has been suggested that the hierarchical organization found in gradients corresponds to the hierarchy present in cognitive processes, reflecting the transformation of sensory input into abstract representations [26]. Therefore, alterations in gradients would point towards disturbances in this hierarchical stream of processing and might reflect disturbances in perception and higher cognitive functions. Recent studies have shown various alterations in brain gradients in patients with depression or psychosis indicating changes in functional integration and segregation compared to healthy controls (HC) [27–30].

In patients with schizophrenia studies report alterations in the visual-to-sensorimotor gradient [27], both the visual-to-sensorimotor and the sensorimotor-to-association gradient [31] or decreased dispersion in multiple brain networks indicating a blurred differentiation between networks [27]. Some studies show alterations in cortical hierarchy in earlier disease stages such as recent-onset psychosis (ROP) [30], while others do not [27]. This leads to the question if alterations only appear with disease progression or already early on in the disease course and possibly already in CHR-P. Interestingly a compression of the sensory-to-association gradient has been found for both schizophrenia and depression, suggesting overlapping and possibly transdiagnostic alterations of the cortical hierarchy [29, 31]. Some initial studies report associations between symptoms and gradient alterations in both patient groups [27–29, 32], including positive, negative and depressive symptoms. However, it remains unclear, if alterations in the hierarchical organization are transdiagnostic, in a way that effects appear in similar regions and share directionality and if they relate to symptoms across disorders. Grouping depression, psychosis and CHR-P, allows us to distinguish general from disorder-specific alterations in hierarchical brain organization.

In addition to clinical associations there is growing evidence that alterations in macroscale brain organization may be linked to microscale neurochemical factors, such as neurotransmitter systems. In depression, associations between gradient alterations and receptor expression related to the dopamine, serotonin, and norepinephrine systems have been reported [33]. Similarly, in schizophrenia, associations have been observed for the serotonin and dopamine systems [34], suggesting that comparable mechanisms may underlie disruptions in hierarchical brain organization across disorders. Moreover, additional neurotransmitter systems, including gamma aminobutyric acid (GABA), glutamate and acetylcholine, have been implicated in the pathophysiology of both disorders [35–37].

In the present study, we aim to investigate if alterations in cortical hierarchy are already present in early phases of depression and psychosis as well as individuals at CHR-P. We specifically aim to address: 1) whether these groups show shared or diagnosis-specific alterations in cortical hierarchical organization, 2) how such alterations relate to clinical and cognitive features, and 3) whether they correspond to spatial patterns of neurotransmitter receptor expression.

## Material and methods

### Data

Here, data from the PRONIA study (Personalized Prognostic tools for early psychosis management, http://www.pronia.eu/) [38] was analyzed. This study has been conducted across 10 sites in Europe and includes HC subjects (*n* = 393), patients with ROP (*n* = 255), patients with recent-onset depression (ROD) (*n* = 229), and individuals with CHR-P (*n* = 247), who underwent resting-state functional magnetic resonance imaging (rs-fMRI). Inclusion and exclusion criteria can be found in the Supplementary Methods.

Prior to study inclusion all participants provided their written informed consent themselves or in case of minor participants, informed consent was provided by their guardians. At each location, the study was approved by the local research ethics committees and registered at the German Clinical Trials Register (DRKS00005042).

Of the 1 124 participants that underwent rs-fMRI, 23 participants were excluded because of motion during the MRI acquisition and 2 because of signal loss. Furthermore, we excluded 22 participants because of missing values for sex or age and all 6 participants from Bari because this small number made correction for site effects impossible. This led to a sample size of 1 071 participants for group analyses. For analyses regarding the association to clinical variables, 18 additional subjects were excluded due to missing data (Supplementary Fig. 1). See Table 1 and Table 2 for demographic and clinical characteristics.

**Table 1:** Characteristics of study groups.

Characteristics	HC (*n* = 376)	ROP (*n* = 243)	ROD (*n* = 212)	CHR-P (*n* = 240)	Full sample (*n* = 1 071)	*F*(3,1 067)/ X^2^(3)	*p*
Sample size per site							
Munich	60^a,b^	87	75	85	307	5.95	0.114
Milan	21	26	16	24	87	2.61	0.456
Basel	56^a,b,c^	25	15	22	118	33.53	<0.001
Cologne	64^a,b,c^	29	42	32	167	18.03	<0.001
Birmingham	41^a,b,c^	14	16	16	87	22.84	<0.001
Turku	51^b,c^	39^b^	20	27	137	16.31	<0.001
Udine	72^a,b,c^	15	23	23	133	61.50	<0.001
Münster	11	8	5	11	35	2.83	0.419
Number of females	219^a^	102^b,c^	118	125	603	16.63	<0.001
Age, mean (SD)	27.96 (6.34)^b^	27.89 (5.65)^b^	28.18 (6.41)^b^	26.03 (5.44)	27.56 (6.06)	6.73	<0.001
Education Years, mean (SD)	15.65 (2.70)^a,b,c^	14.29 (7.82)	14.46 (3.06)^b^	13.49 (2.70)	14.62 (4.64)	11.75	<0.001
CPZE, mean (SD)^1^	0 (0)^a,b,c^	153.00 (156.97)^b,c^	18.11 (66.21)	22.79 (55.75)	42.69 (102.82)	179.10	<0.001
Mean framewise Displacement, mean (SD)	0.16 (0.09)^b,c^	0.18 (0.11)	0.19 (0.11)	0.18 (0.11)	0.18 (0.10)	3.67	0.012

*CHR-P* clinical high-risk, *HC* healthy controls, *ROD* recent-onset depression, *ROP* recent-onset psychosis.

Subscripts indicate significant differences after post-hoc t-test at the pFDR < 0.05 level after false discovery rate correction for multiple comparisons; a vs. ROP; b vs. CHR-P; c vs. ROD. ^1^ 7 ROP patients with depot medication were excluded.

**Table 2:** Characteristics of participants included in analysis with clinical symptoms.

Characteristics	ROP (*n* = 234)	ROD (*n* = 207)	CHR-P (*n* = 236)	Full sample (*n* = 677)		*F*(2, 674)/ X^2^(2)	*p*
Number of females	99^a^	114	122	334	7.87		0.020
Age, mean (SD)	27.92 (5.67)^b^	28.34 (6.39)^b^	26.06 (5.40)	27.40 (5.89)	9.99		<0.001
Education Years, mean (SD)	14.41 (7.96)	14.51 (3.07)	13.51 (2.71)	14.12 (5.24)	2.51		0.082
BDI, mean (SD)	19.27 (10.47)^a,b^	24.51 (11.69)	24.42 (10.88)	22.67 (11.26)	17.05		<0.001
PANSS Positive, mean (SD)	19.23 (5.86)^a,b^	8.53 (2.45)^b^	11.78 (3.59)	13.36 (6.17)	370.76		<0.001
PANSS Negative, mean (SD)	15.65 (7.54)^a,b^	12.79 (5.27)	13.81 (6.67)	14.13 (6.70)	10.71		<0.001
PANSS General, mean (SD)	34.61 (10.51)^a,b^	28.17 (7.59)	29.27 (8.13)	30.78 (9.30)	34.15		<0.001
GF-S, mean (SD)	5.63 (1.53)^a,b^	6.34 (1.33)	6.17 (1.47)	6.04 (1.48)	14.95		<0.001
GF-R, mean (SD)	5.10 (1.80)^a,b^	6.09 (1.78)	5.72 (1.76)	5.62 (1.82)	17.72		<0.001
GAF-DI past month, mean (SD)	44.82 (13.31)^a,b^	55.24 (14.88)^b^	52.59 (13.06)	50.71 (14.40)	35.01		<0.001
GAF-S past month, mean (SD)	40.94 (13.69)^a,b^	54.19 (12.74)	52.17 (11.08)	48.91 (13.82)	73.67		<0.001
Cognition Total, mean (SD)	−0.67 (0.82)^a,b^	−0.16 (0.63)	−0.25 (0.59)	−0.37 (0.72)	34.85		<0.001
WM, mean (SD)	−0.63 (0.99)^a,b^	−0.22 (0.84)	−0.14 (0.83)	−0.33 (0.92)	20.18		<0.001
Social Cognition, mean (SD)	−0.41 (1.14)^a,b^	−0.08 (0.90)	−0.19 (0.90)	−0.23 (1.00)	6.76		0.001
Verbal Learning, mean (SD)	−0.50 (1.13)^a,b^	−0.10 (0.92)	−0.17 (0.95)	−0.26 (1.02)	10.30		<0.001
SoP, mean (SD)	−0.80 (0.88)^a,b^	−0.23 (0.72)	−0.35 (0.66)	−0.47 (0.80)	36.08		<0.001
Attention, mean (SD)	−0.98 (1.96)^a,b^	−0.15 (1.68)	−0.42 (1.70)	−0.53 (1.82)	12.51		<0.001
VisDys, mean (SD)	3.96 (7.92)^a^	0.71 (2.03)^b^	3.81 (6.16)	2.91 (6.18)	20.04		<0.001

*BDI* beck´s depression inventory, *CHR-P* clinical high-risk, *CPZE* chlorpromazine equivalent, *GAF-DI* global assessment of functioning disabilities, *GAF-S* global assessment of functioning symptoms, *GF-R* global functioning role, *GF-S* global functioning social, *HC* healthy controls, *ROD* recent-onset depression, *ROP* recent-onset psychosis, *SoP* speed of processing, *VisDys* visual dysfunctions, *WM* working memory.

Subscripts indicate significant differences after post-hoc *t*-test at the *p*_*FDR*_ < 0.05 level after false discovery rate correction for multiple comparisons; ^a^ vs. ROD. ^b^ vs. CHR-P.

### Clinical and neuropsychological assessment

The Schizophrenia Proneness Instrument (SPI-A) [39] and Structured Interview for Psychosis-Risk Syndromes [40] were used to assess CHR-P status. SPI-A items related to visual dysfunctions (VisDys) were used to calculate a VisDys score as described before [16]. Further positive, negative and general psychotic symptoms were assessed by the Positive and Negative Syndrome Scale (PANSS) [41]. Depressive symptoms were assessed via self-report using the Beck’s Depression Inventory–II [42]. The Global Functioning: Role Scale (GF-Role), the Global Functioning: Social Scale (GF-Social) [43] and the Global Assessment of Functioning (GAF) [44] were used to assess functioning.

Five cognitive domains were measured by seven tests. The domains included social cognition, working memory, speed of processing, verbal learning and attention, building together the global cognition score. More information on the used tests and the computation of the scores can be found in the Supplementary Methods.

For analyses regarding the association to clinical variables, participants with more than 30% missing data points in the clinical scales were excluded. For participants with fewer missing values the values were imputed by the median of the corresponding group.

### MRI acquisition and preprocessing

T1 reference images were acquired using a multi-echo MPRAGE sequence. At all sites, rs-fMRI scans were acquired using echo planar imaging sequences with 200 volumes and a repetition time of 3 s, resulting in a duration of 603 s. The participants were instructed to keep their eyes open throughout the scan. As acquisition parameters differed between acquisition sites, details of the acquisition sites can be found in the Supplementary Tables 1 and 2. The data was preprocessed with a pipeline developed by the PRONIA consortium [45].

For preprocessing the Statistical Parametric Mapping software (SPM, version 12-6685; http://www.fil.ion.ucl.ac.uk/spm) and the Resting State fMRI data analysis Toolkit (REST, version 1.8; http://www.restfmri.net/) were used. The first 8 volumes were discarded, and images were slice-time corrected and realigned to the first volume. The functional maps were co-registered to the T1-weighted images, resliced, and normalized to the common Montreal Neurological Institute space. Furthermore, white matter, cerebrospinal fluid and the Friston 24 motion parameters, comprising translation and rotation in three directions, temporal derivatives and their quadratic terms [46], were regressed out as covariates. Finally, Smoothing was applied, motion was corrected using time series de-spiking, and scans were detrended. For our analysis participants with a mean framewise displacement over 0.50 mm for more than 38.5% of volumes were excluded [47]. This is in line with the preprocessing in other studies from the PRONIA sample [45, 48].

### Functional gradient computation

**Fig. 1:**
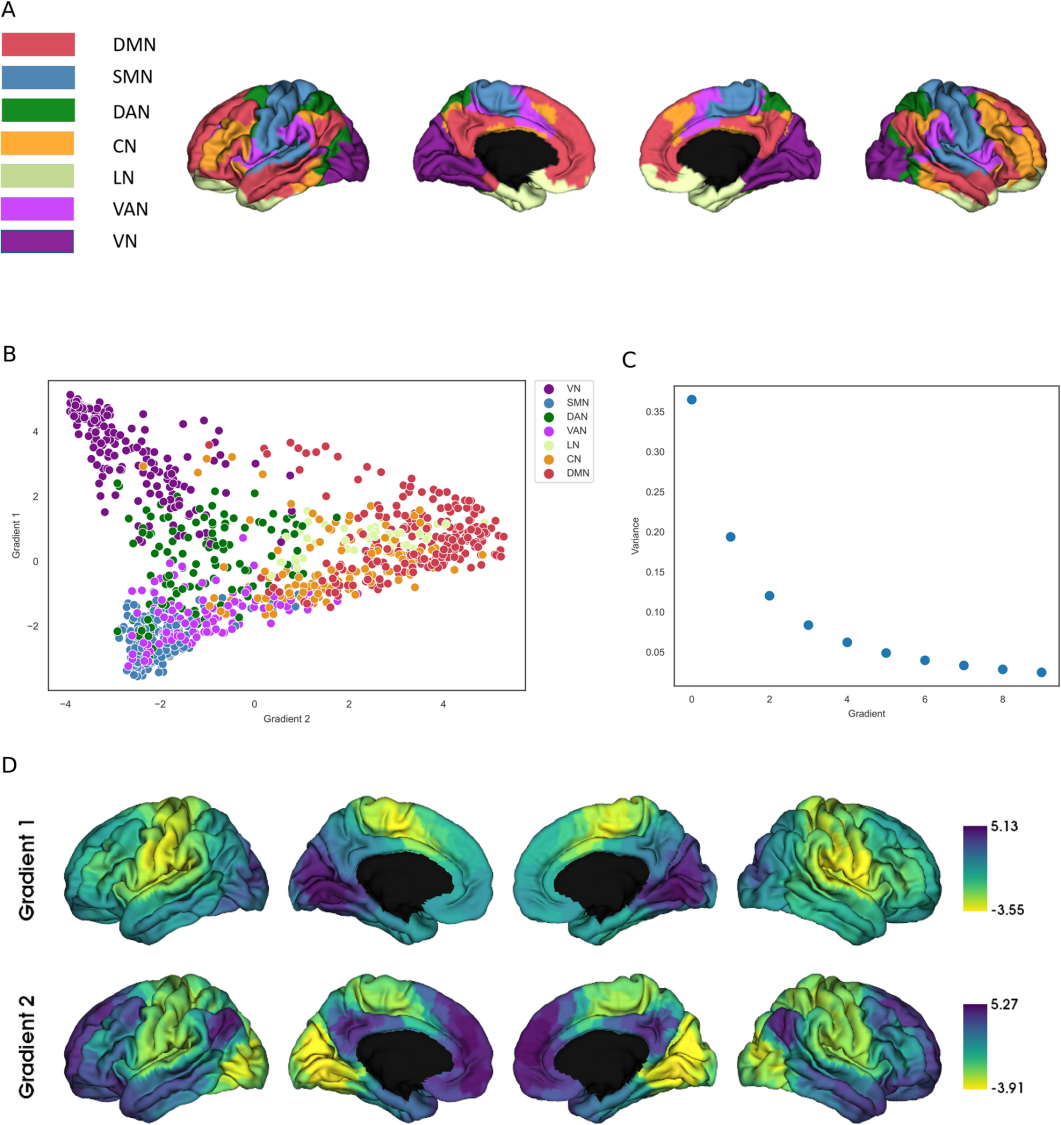
Gradient structure across groups. **A** Schaefer 7 network parcellation. **B** Average gradient scores in the two-dimensional gradient space. **C** Mean variance explained by gradients. **D** Spatial topography of first and second gradient. CN control network; DAN dorsal attention network; DMN default mode network; LN limbic network; SMN somatomotor network; VAN ventral attention network; VN visual network.

In accordance with previous studies [27, 30], we used the Schaefer parcellation [49] with 1 000 parcels, which includes annotations of the 7 Yeo networks [50] (Fig. 1). This allowed us to investigate network specific alterations in the DAN, the CN, the DMN, the LN, the VN, the somatomotor network (SMN) and the ventral attention network (VAN). Pearson correlation was used to compute a FC matrix for every participant, which was then standardized using the Fisher z-transformation. The gradients were derived with the Python implementation of the BrainSpace toolbox [24]. In line with previous research [27, 51], the FC matrices were thresholded to retain the strongest 10% of connections of each region. Next an affinity matrix was computed using cosine similarity and decomposed into a set of principal eigenvectors using diffusion mapping as a dimensionality reduction method. These eigenvectors serve to describe the underlying gradients [24] (see Supplementary Fig. 2 for analysis pipeline). Individual gradients were aligned to a reference gradient derived from the mean FC from the HC group in our study sample via Procrustes rotation [52] to ensure interpretability (see Supplementary Fig. 3 for reference gradients). The gradients were then named according to the regions they were anchored in. In order to account for differences in MRI scanners between sites, we employed ComBat [53] on the gradient scores and derived metrics (see Supplementary Methods for details). This method has been shown to be efficient for fMRI data [54].

### Functional gradient differences between HC and clinical groups

Differences in scores of the first two gradients between each clinical group and HC were explored regionally using the surface-based linear model from the BrainStat toolbox [55]. Age, sex and mean framewise displacement (mean FD) were included in the models as covariates. P-values were corrected for multiple comparisons over the 1 000 regions by the false discovery rate (FDR) correction [56]. Because the data were not normally distributed, group differences in mean network values between each clinical group and HC were assessed using Mann-Whitney-U-tests, which do not assume normality or equal variances. Prior to testing, the effects of age, sex, and mean FD were regressed out. Mean framewise displacement was included to account for potential motion effects. Again p-values were corrected for multiple comparisons across the 7 networks and multiple groups (number of tests per gradient: 28) using FDR-correction.

### Group differences in functional differentiation

We assessed the range of gradient scores as a measure for functional differentiation by subtracting the minimum from the maximum gradient value of each subject. In a next step, we investigated network-specific functional differentiation by deriving measures of within- and between-network dispersion [51] in the two-dimensional gradient space. For this, the central region of each network was computed as the median of all regions in that network. Each region is represented by two coordinates, with the first coordinate indicating the value of that region on the first gradient and the second coordinate representing the value of this region on the second gradient. Within-network dispersion was calculated as the mean Euclidean distance between this central region and all other regions belonging to that network. Between-network dispersion was calculated as the Euclidean distance between the central regions of different networks, leading to 7 within-network dispersion scores and 21 between-network dispersion scores per participant. Group differences in these measures were also evaluated using Mann-Whitney-U-tests. All p-values regarding group differences involving multiple comparisons were corrected using FDR-correction across all comparisons within this measure (range gradient 1 and 2: 4 each, within-network dispersion: 28, between-network dispersion: 84).

### Association of Gradient Alterations with Clinical Measures and Receptor Expression Maps

We used correlation analyses to explore associations between gradient dispersion metrics and network gradient values and clinical variables over all clinical groups. All p-values were then FDR-corrected within the specific gradient measure (number of associations: gradient 1, 2 and within-network dispersion: 105 each, between-network dispersion: 315). To test, if relationships between variables are more complex, than captured by simple correlation analysis, we performed canonical correlation analyses (CCA) (for details see Supplementary Methods).

We furthermore evaluated associations between *t*-maps of HC and clinical groups and publicly available receptor expression maps from positron emission tomography scans using the neuromaps toolbox [57]. As this has not been extensively studied and many neurotransmitter systems are involved in the diseases we exploratively used all available receptor maps covering receptors of nine neurotransmitter systems including receptors/transporters for acetylcholine, dopamine, cannabinoid, opioid, glutamate, serotonin, GABA, norepinephrine and histamine (see Supplementary Table 3 for details). Maps for the same receptor types were averaged to improve signal-to-noise ratio (see Supplementary Fig. 4 for correlations). Significance was tested based on 10 000 spin permutations [58] to account for spatial autocorrelation. To account for multiple comparisons, results were FDR-corrected within each diagnostic group and gradient over the different receptor types (number of tests in each group: 21).

## Results

### Functional gradient differences between hc and clinical groups

We found the first gradient in our data to span sensorimotor and visual areas and the second gradient to span the DMN and sensorimotor areas (Fig. 1). Neither for gradient 1 nor for gradient 2 did the explained variances differ between groups, allowing us to compare gradients between groups. The visual-to-sensorimotor gradient explained on average 36.25% (*SD* = 7.90) of variance for HC, 36.32% (*SD* = 8.41) for CHR-P, 37.41% (*SD* = 8.26) for ROP and 36.09% (*SD* = 7.63) for ROD. The sensory-to-association gradient explained on average 19.50% (*SD* = 4.26) of variance for HC, 19.55% (*SD* = 3.93) for CHR-P, 19.27% (*SD* = 4.18) for ROP and 19.09% (*SD* = 3.60) for ROD.

**Fig. 2:**
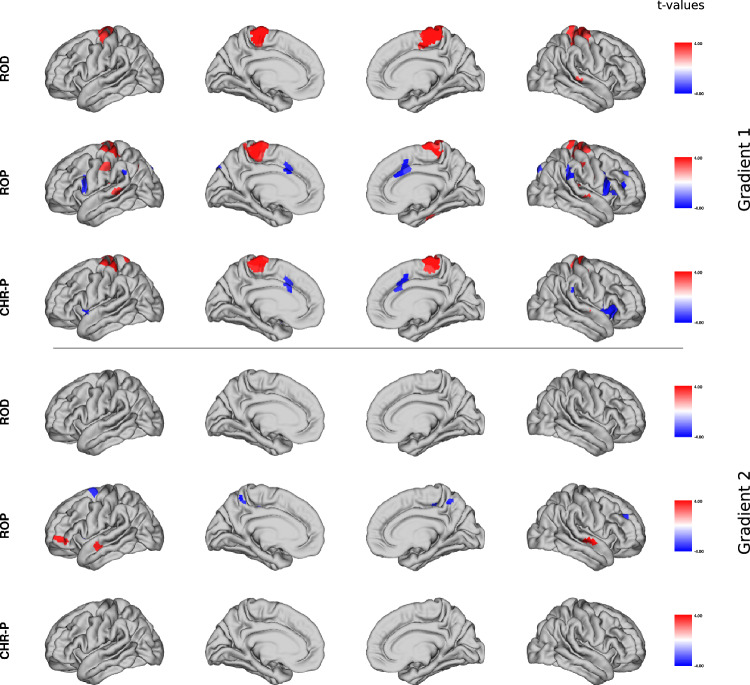
Regional differences between patient groups and healthy controls. *T*-values of regions that significantly differ in their gradient value between HC and patient groups after FDR-correction with *p*_*FDR*_ < 0.05; models were controlled for age, sex and mean framewise displacement; higher/lower values in patient groups are presented in red/blue. CHR-P clinical high-risk; ROD recent-onset depression, ROP recent-onset psychosis.

All three clinical groups showed higher scores in the SMN in the visual-to-sensorimotor gradient compared to HC (*t* > 3.00, all *p*_FDR_ < 0.048) except for CHR-P individuals who showed lower values in one single region of the SMN (*t* = −3.90, *p*_FDR_ = 0.010). For ROP patients, significant differences also appeared in regions belonging to all other networks (−5.04<*t* < 4.05, all *p*_FDR_ < 0.046) but most prominent were regions of the VAN, with ROP patients showing lower values than HC (all *t* < −3.20, all *p*_FDR_ < 0.045). CHR-P individuals also exhibited significantly lower values in regions of the VAN (all *t* < −3.27, all *p*_FDR_ < 0.049) and additionally showed some significant differences in a few regions of the LN (−3.32<*t* < 3.29, all *p*_FDR_ < 0.046) and significantly lower values in a few regions of the CN and DMN (all *t* < −3.30, all *p*_FDR_ < 0.046). ROD patients showed additionally significantly higher values in one region of the DAN (*t* = 3.19, *p*_FDR_ = 0.030). Alterations in the sensory-to-association gradient were less pronounced. ROD patients only showed significant differences in one region, belonging to the CN (*t* = −4.12, *p*_FDR_ = 0.043), with values being lower than in HC. ROP patients showed lower values in some regions of the DAN and VAN (all *t* < −3.41, all *p*_FDR_ < 0.043), higher values in a few regions of the CN and DMN (all *t* > 3.69, all *p*_FDR_ = 0.034) and mostly higher values in regions of the SMN (−3.53<*t* < 4.81, all *p*_FDR_ < 0.038). CHR-P individuals showed no significant differences in the sensory-to-association gradient. Statistical differences in the gradients are displayed on the cortical surface in Fig. 2.

**Fig. 3:**
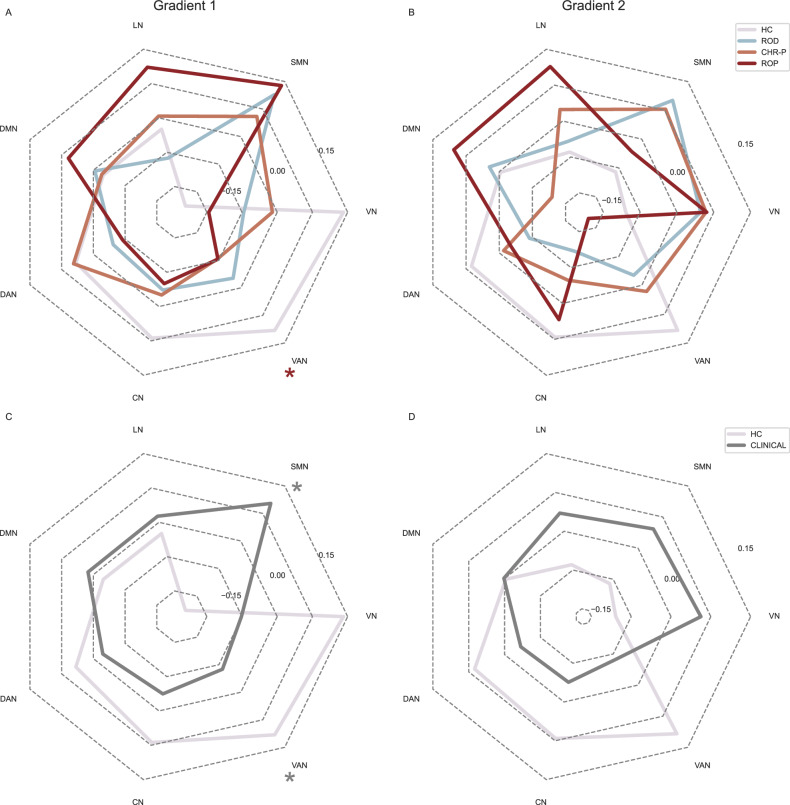
Differences between patient groups and healthy controls in mean network values. Gradient values are displayed after regressing out age, sex and mean framewise displacement. Colors of stars indicate which groups differ from HC after FDR-correction with *p*_FDR_ < 0.05. **A** Gradient 1 for HC vs. clinical groups. **B** Gradient 2 for HC vs. clinical groups. **C** Gradient 1 for HC vs. clinical groups combined. **D** Gradient 2 for HC vs. clinical groups combined. CHR-P clinical high-risk; CN control network; DAN dorsal attention network; DMN default mode network; HC healthy controls; LN limbic network; CLINICAL = ROP, ROD and CHR-P combined; ROD recent-onset depression; ROP recent-onset psychosis; SMN somatomotor network; VAN ventral attention network; VN visual network.

The analysis of mean network values confirmed the findings of the regional analysis, which indicated that the focus of alterations was on the first gradient. For the visual-to-sensorimotor gradient we found significantly lower scores in the VAN for ROP patients (*U* = 52 930, *p*_*FDR*_ = 0.022, η^2^ = 0.0131) and clinical groups in general (*U* = 149 052, *p*_*FDR*_ = 0.004, η^2^ = 0.0117) compared to HC. CHR-P individuals also showed lower values in the VAN, but this difference did not survive FDR-correction (*U* = 51 736, *p*_*FDR*_ = 0.052, η^2^ = 0.0134). Furthermore, we found higher scores in the SMN when comparing all clinical groups to HC (*U* = 113 120, *p*_*FDR*_ = 0.008, η^2^ = 0.0168). For ROD no differences survived FDR-correction. We did not find any significant differences regarding the sensory-to-association gradient on the network-level after FDR-correction (Fig. 3).

### Group differences in functional differentiation

Overall, clinical groups showed a smaller range of the visual-to-sensorimotor gradient compared to HC (*U* = 143 701, *p*_FDR_ = 0.021, η^2^ = 0.0041). This effect was also present when comparing only ROD to HC (*U* = 45 508, *p*_FDR_ = 0.017, η^2^ = 0.0083). There were no differences between any clinical group and HC in the sensory-to-association gradient.

**Fig. 4:**
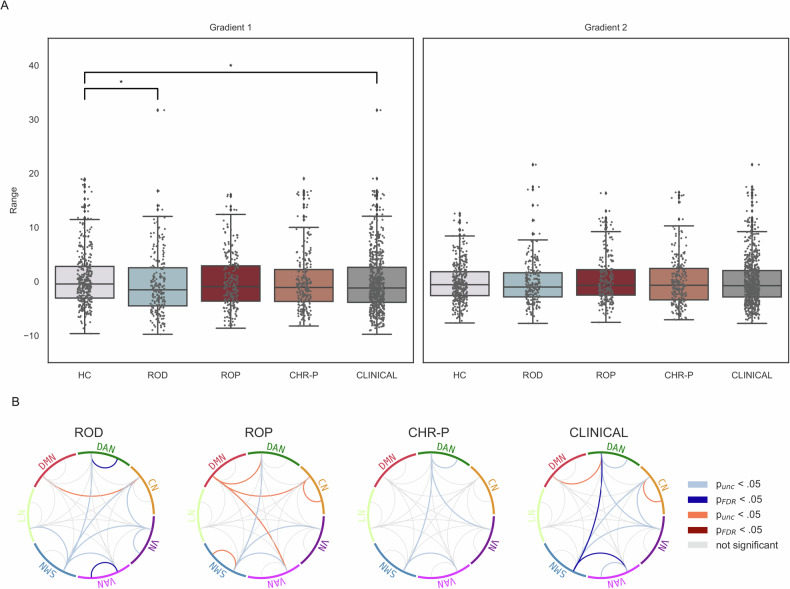
Functional Differentiation. **A** Range of gradients. Whiskers indicate interquartile range. Asterisks indicate significant differences after FDR-correction; * *p*_FDR_ < 0.05. **B** Between-group differences in within- and between-network dispersion. Grey lines indicate non-significant differences. Red/dark blue lines indicate significantly higher/lower values for the respective patient group with *p*_*FDR*_ < 0.05 after FDR-correction. Orange/light blue lines indicate significantly higher/lower values for the respective patient group before FDR-correction. CHR-P clinical high-risk; CN control network; DAN dorsal attention network; DMN default-mode network; HC healthy control; LN limbic network; CLINICAL = ROP, ROD and CHR-P combined; ROD recent-onset depression; ROP recent-onset psychosis; SMN somatomotor network; VAN ventral attention network; VN visual network.

Analyses of the network dispersion revealed significant differences within networks only for ROD patients in the DAN (*U* = 46 729, *p*_FDR_ = 0.014, η^2^ = 0.0119) and VAN (*U* = 46 296, *p*_FDR_ = 0.030, η^2^ = 0.0112), with ROD patients exhibiting lower scores than HC. Analysis of between-network dispersion showed lower scores for the dispersion in clinical groups between SMN and VAN (*U* = 148 271, *p*_FDR_ = 0.022, η^2^ = 0.0144) and between SMN and DAN (*U* = 147 291, *p*_FDR_ = 0.047, η^2^ = 0.0123) as compared to HC after FDR-correction. Differences in separate clinical group analyses did not survive FDR-correction but indicated numerically lower scores in all clinical groups as compared to HC (Fig. 4).

Median values and interquartile ranges for all gradient measures can be found in Supplementary Table 4.

### Association of gradient alterations with clinical measures

**Fig. 5:**
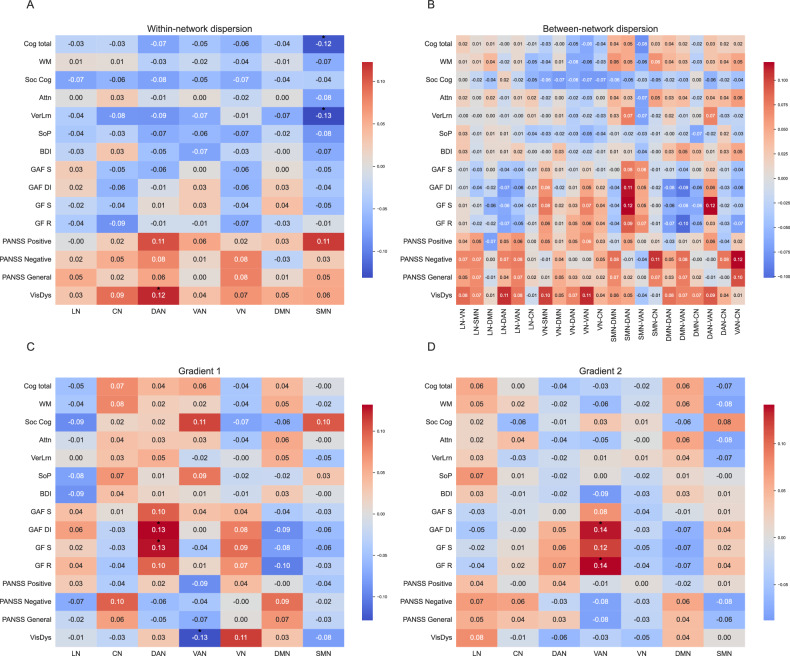
Association of clinical variables with gradient measures. Asterisks indicate significant correlations after FDR-correction; * *p*_FDR_ < 0.05. **A** Correlations of within-network dispersion with clinical variables. **B** Correlations of between-network dispersion with clinical variables. **C** Correlations of mean-network values of gradient 1 with clinical variables. **D** Correlations of mean-network values of gradient 2 with clinical variables. Attn attention; BDI Becks Depression Inventory; CN control network; Cog total cognition total score; DAN dorsal attention network; DMN default-mode network; GAF DI Global Assessment of Functioning Disability; GAF S Global Assessment of Functioning Symptoms; GF R Global Functioning Role Scale; GF S Global Functioning Social Scale; LN limbic network; Soc Cog social cognition; SoP speed of processing; SMN somatomotor network; VAN ventral attention network; VerLrn verbal learning; VisDys visual dysfunctions; VN visual network; WM working memory.

We found small correlations between clinical measures of functioning and mean network values of the visual-to-sensorimotor and the sensory-to-association gradient in the attentional networks. Mean values of the DAN of the visual-to-sensorimotor gradient correlated significantly positive with GF-S (*r* = 0.13, *p*_FDR_ = 0.030) and GAF-DI (*r* = 0.13, *p*_FDR_ = 0.030) scores and the mean values of the VAN of the sensory-to-association gradient correlated significantly positive with GF-R (*r* = 0.14, *p*_FDR_ = 0.018) and GAF-DI (*r* = 0.14, *p*_FDR_ = 0.018) scores. Moreover, visual dysfunctions were negatively associated with mean values of the VAN of the visual-to-sensorimotor gradient (*r* = −0.13, *p*_FDR_ = 0.030) and positively with the within-network dispersion of the DAN (*r* = 0.12, *p*_FDR_ = 0.049). Within-network dispersion of the SMN was negatively associated with verbal learning (*r* = −0.13, *p*_FDR_ = 0.049) and total cognition scores (*r* = −0.12, *p*_FDR_ = 0.049) (Fig. 5). For exact FDR corrected p-values see Supplementary Tables 5 – 8. We repeated these analyses for diagnostic groups separately, after which no associations remained significant (Supplementary Figs. 6–8). However, the pattern of correlations remained similar (Supplementary Fig. 9). CCA revealed multiple significant modes, with the first one showing a medium association between variable sets (*r* = 0.44, *p*_FDR_ = 0.007). However only clinical variables loaded significantly on the canonical modes (Supplementary Results).

### Association of gradient alterations with receptor expression maps

**Fig. 6:**
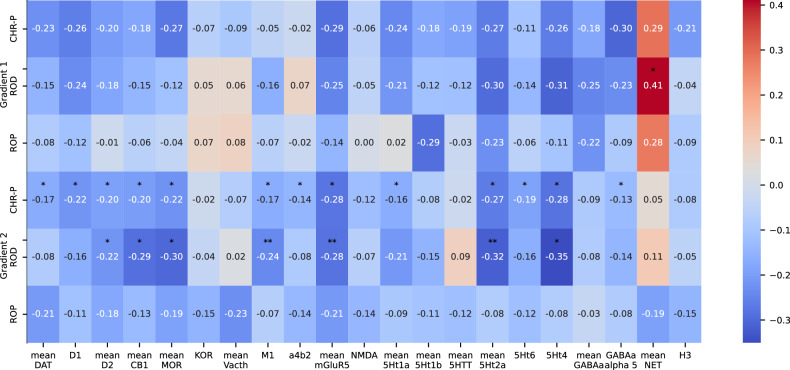
Association of receptor maps with case-control differences in gradients. Asterisks indicate significant correlations after FDR-correction; * *p*_spin-FDR_ < 0.05; ** *p*_spin-FDR_ < 0.01. CHR-P clinical high-risk; ROD recent-onset depression; ROP recent-onset psychosis.

We identified spatial concordances between the gradient t-maps of clinical groups and receptor expression maps. Most pronounced were negative associations for ROD patients between serotonin receptors and variations in the sensory-to-association gradient (*r* < −0.31, *p*_FDR_ < 0.014). CHR-P individuals showed negative associations with the same receptor maps as ROD patients, but also to additional receptor maps (all *r* < −0.13, all *p*_FDR_ < 0.046). No significant associations were identified for ROP patients. The only significant association for the visual-to-sensorimotor gradient was found for ROD patients with the norepinephrine transporter (NET) (*r* = 0.41, *p*_FDR_ = 0.031) (Fig. 6). Exact FDR-corrected p-values for all receptors and gradients can be found in Supplementary Table 9.

### Sensitivity analyses

To examine potential medication effects, we correlated the VAN alterations observed in ROP patients with daily CPZE, but no significant relationship was detected (Supplementary Fig. 10).

We also tested the influence of choosing a different threshold before computing the affinity matrix, retaining the strongest 20% of connections. That switched the order of the gradients, but there was still a high correspondence between the visual-to-sensorimotor gradients and between the sensory-to-association gradients (*r* = 0.925) (Supplementary Fig. 11).

We further examined the impact of the alpha parameter in the diffusion map embedding and found that embeddings derived from different alpha values remained highly correlated (*r* > 0.974) (Supplementary Fig. 12).

We additionally performed GLM analyses with all groups in one model. Overall results remained consistent with some differences in significance after FDR-correction. Results including ß coefficients and confidence intervals for the clinical groups can be found in Supplementary Figs. 13–16.

## Discussion

In this study we investigated disease-specific and transdiagnostic alterations of hierarchical brain organization in functional brain gradients of rs-FC and their association to clinical symptoms. To the best of our knowledge we are the first to have examined this in CHR-P.

We found a similar gradient structure as reported by other studies [27, 29, 31]: One gradient represented the commonly found sensory-to-association axis and the other gradient spanned visual and sensorimotor areas. The order of gradients is not always consistent across studies. Although the sensory-to-association gradient typically explains the most variance some studies report the reverse ordering with the visual-to-sensorimotor gradient appearing first [23, 24]. This was also observed in our study. However, as the gradients still represent the commonly found axes of variation and are anchored in the same regions, interpretability is still given.

For all groups we mainly found alterations in the visual-to-sensorimotor gradient. The networks that showed the most alterations were the SMN for all clinical groups and the VAN for CHR-P and ROP. In the sensory-to-association gradient, alterations were less pronounced and mainly shown by ROP patients again in the SMN and VAN. The pronunciation of alterations in the visual-to-sensorimotor gradient is supported by a recent study of gradients in schizophrenia [27] and aligns with findings of altered visual-to-sensorimotor rs-FC in psychosis [59]. Another study found similar patterns of alterations to our ROP group in the sensorimotor-to-visual gradient in schizophrenia, although differences were more widespread [31], which might be explainable by the progressed illness stage in their sample.

Our findings in the VAN are in line with previous studies, that showed aberrant connectivity of this network in psychosis and CHR-P [13, 15]. As the effect for clinical groups in general seems to be driven by the ROP and CHR-P individuals, these alterations in the VAN seem to be specific for the psychosis spectrum. This is consistent with disturbances in salience detection and assignment in psychotic symptoms like delusions and hallucinations [60], which are less common in depression.

Alterations in the SMN that are shown by all clinical groups imply the involvement of the SMN in transdiagnostic processes. The SMN has already been identified as a transdiagnostic network [61] and shows alterations across psychiatric disorders [18]. Previous studies investigating functional gradients have also found alterations of basic networks in depression [29] and psychosis [31]. It has been suggested that these results imply a “bottom-up dysregulation” [31], as alterations in the SMN can be found even when networks of higher functions remain intact [62].

The found alterations in the SMN and VAN suggest a disruption in the flow of bottom-up sensory and attentional information and are in line with findings of a disturbed integration of sensory information in psychosis [63], supporting the theory of disturbed bottom-up information processing leading to impairment of higher functional networks. Specifically, alterations in the VAN, which plays a key role in detecting salient stimuli, may impair the propagation of sensory signals to higher-order cognitive networks. These results support models proposing that impaired lower-level processing cascades into broader network dysfunction.

In contrast to our results, a prior study did not find alterations in psychosis, but only in chronic schizophrenia, concluding that the hierarchical organization seems to change only with progression of illness [27]. A reason for this may be their less impaired sample. Compared to their early psychosis sample, our ROP sample showed higher positive and negative symptom severity and even our CHR-P sample had a higher negative symptom score. Additionally, our sample size may have allowed us to detect subtle alterations, that might go undetected in a smaller sample.

### Functional differentiation

We found lower functional differentiation and higher integration within networks in ROD, manifesting in a lower range in the sensorimotor-to-visual gradient and lower within-network dispersion in the attentional networks. Lower within-network dispersion, indicating more homogeneous FC profiles in these networks, has been associated with a higher nodal degree from a graph-theoretical perspective, reflecting stronger internal integration of network nodes [28]. Within the gradient framework, such changes may reflect altered integration of networks within an otherwise preserved cortical hierarchy. Consistent with recent work, alterations in gradient-derived metrics are often interpreted as subtle shifts in the positioning of networks along hierarchical functional axes rather than large-scale disruptions of cortical topology [22]. A lower range of gradient values in depression has been reported previously in the sensory-to-association gradient suggesting reduced functional segregation [29]. However, the visual-to-sensorimotor gradient examined here was not investigated in these studies. These findings suggest a shift between segregation and integration, a core principle of efficient brain organization [64].

While regional and network analyses revealed similar alterations for psychosis and CHR-P, analysis of functional differentiation showed similarities between all clinical groups in the form of lower between-network dispersion that resulted in an overall effect for clinical groups. This indicates more similar FC profiles and is in line with findings of transdiagnostic de-differentiation in network organization in schizophrenia and depression [18]. Regarding the range, there was also an effect for clinical groups combined, driven by depression and CHR-P, suggesting similarities between these groups. This links with the clinical practice as depression is the most prevalent comorbidity in individuals with CHR-P [65] and many CHR-P individuals develop an affective disorder [66]. Therefore, alterations in functional differentiation in CHR-P individuals might reflect parts of the high-risk state other than the psychotic aspect.

### Association with clinical symptoms

We observed modest associations between gradient scores in attentional networks and clinical measures of functioning, suggesting that alterations in hierarchical brain organization may relate more to complex clinical measures rather than to single symptoms. Within-network dispersion in the DAN and network values of the VAN in the sensorimotor-to-association gradient were weakly associated with VisDys. This suggests that additionally to the CN, that has been found to be predictive of VisDys in the PRONIA sample [16], disturbances in the VAN during hierarchical processing might be involved in VisDys symptoms. Additionally, within-network dispersion in the SMN was associated with cognition, which might seem striking. Indeed alterations of the SMN have been related to cognitive dysfunction before [61]. Nevertheless, correlations are rather small, which might be due to small effect sizes of our results regarding the hierarchical organization. Furthermore, this and the lack of significant associations in diagnosis-specific analyses might indicate that gradient changes may reflect trait vulnerability or disease progression rather than state effects or clinical symptoms. Longitudinal studies are needed to clarify this.

### Association to receptor maps

We discovered spatial associations between multiple receptor maps and gradient abnormalities in ROD and CHR-P. Most associations were found for the sensory-to-association gradient, suggesting that neurotransmitter systems are mainly involved in disturbances of the axis separating unimodal from trans-modal areas, even if these alterations themselves are not significant. Our findings in ROD align with previous studies, that also found negative associations between case-control-differences and expression of 5Ht2a and 5Ht1b and positive associations with NET [33]. Dopaminergic and serotonergic systems have been previously linked to mood disorders [67] and NET has been associated with functions in unimodal regions [68], potentially explaining its unique associations in the visual-to-sensorimotor gradient. These three systems are involved in regulating information processing loops of attentional and sensory processes [69], explaining their association to disruptions in hierarchical brain organization. While associations to NET seem to be specific to depression, other associations in ROD show an overlap to CHR-P, which shows associations with multiple receptor systems. This may reflect the heterogeneity of CHR-P and its frequent comorbidities. The overlap between receptor maps associated with CHR-P and ROD points into the same direction, highlighting similarities between those two populations. ROP showed no significant association to receptor maps despite more severe symptoms, suggesting that these alterations may not directly relate to symptom severity. Importantly, these analyses only demonstrate spatial covariation and do not allow conclusions about causality. Longitudinal investigations are required to identify if changes in neurotransmitter systems cause or follow changes in hierarchical brain organization.

### Limitations

First, we used a parcellation-based rather than a vertex-based approach, which could have influenced the gradients. Nevertheless, highly granular functional parcellation schemes such as the Schaefer parcellation, show strong correspondence with Mesulam´s [26] cortical hierarchy [24].

Second, in the context of multisite MRI data, the issue of site-effects represents a persistent challenge. We chose ComBat as a correction method, as it has often been used for fMRI data. Nevertheless, this could potentially have had an effect on results as it is an artificial way of harmonizing data.

Third, our sample only includes early disease stages, which could explain the small effect sizes and weak associations to clinical symptoms. Even though our findings support the notion that alterations in psychosis might become more pronounced with progression of illness, our study is cross-sectional.

Fourth, our study uses a lower temporal fMRI scan resolution than some other gradient studies [27], which could affect the stability of FC estimates. However, many gradient studies have used similar repetition times and even shorter scan durations [28, 29, 33, 51]. Although longer scan durations are recommended to improve gradient discriminability, we focused on the first two gradients, which show relatively high discriminability even with shorter scans [70]. As our findings in the visual-to sensorimotor gradient are in line with previous findings with longer scans [27], our results are unlikely to be driven by these parameters.

### Conclusion

In summary, we showed that the hierarchical organization of the brain is already disturbed in early disease stages, with the SMN and attentional networks exhibiting the greatest involvement. Our findings suggest that functional differentiation and gradient alterations of the SMN seem to be more general markers of psychiatric disease, while gradient alterations in the VAN seem to be more specific markers for the psychosis spectrum. In general, the findings point towards disturbed integration of bottom-up sensory input and attentional processes in psychiatric disorders. Our study suggests that alterations are already present in early disease stages. Future research should further investigate the role of the SMN in transdiagnostic disease processes and examine the progression of disturbances in the hierarchical organization in a longitudinal sample.

## Supplementary information

Supplementary Materials

## Data Availability

Code is available upon request.
