# Supplementary material for: Disrupted hierarchical functional brain organization in affective and psychotic disorders: insights from functional brain gradients
Source: Transl Psychiatry. 2026 Jul 29;16:384. doi: 10.1038/s41398-026-04206-z (PMC13421452; doi:10.1038/s41398-026-04206-z)
Supplement: Supplementary file 1 — Supplementary Materials [file 41398_2026_4206_MOESM1_ESM.docx]

**Supplementary Materials**

**Supplementary Methods**

**Inclusion and exclusion criteria**

To be included in the study, participants had to be between 15 and 40 years old with sufficient language skills for participation and had to have the capacity to give their informed consent. Exclusion criteria were the following: an IQ below 70, current or past head trauma with loss of consciousness (> 5 minutes), current or past known neurological or somatic disorders that could potentially affect structure or functioning of the brain, current or past alcohol dependence, polysubstance dependence within the past 6 month or medical indications against MRI. Recent-onset depression (ROD) and Recent-onset psychosis (ROP) criteria were assessed by the structured clinical interview for DSM-IV disorders (SCID) (1). ROP patients were included if they had a psychotic episode according to the DSM-IV-TR (Diagnostic and Statistical Manual of Mental Disorders, Text Revision) criteria for a lifetime affective and nonaffective psychotic episode within the past 3 months. In accordance with that ROD patients had to fulfill the DSM-IV-TR criteria for a lifetime major depressive episode within the past 3 months. For both groups the onset of the disease had to be within the last 24 months. Furthermore, ROD as well as ROP subjects were excluded if they were on an antipsychotic medication for longer than 90 days or above the minimum dosage of the first-episode psychosis range of DGPPN S3 Guidelines (2). To be included in the clinical high-risk for psychosis (CHR-P) group, participants either needed to fulfill ultra-high-risk criteria, which were assessed by the Structured Interview for Psychosis-Risk Syndromes (3) or the cognitive disturbances (COGDIS) criteria, assessed by the Schizophrenia Proneness Instrument (SPI-A) (4); in addition to the antipsychotic medication related exclusion criterion described above, an antipsychotic medication for more than 30 days (cumulative number of days) at or above the same minimum dosage range let to exclusion.

Additional exclusion criteria for healthy controls (HC) were Current or past DSM-IV-TR Axis-I disorder (not including nicotine dependency); CHR-P criteria positive (life time); Intake of psychopharmacological substances or illegal drugs for more than 5 days per year and/or during the past month prior examination; Affective or non-affective psychosis or major affective disorder (Major Depressive Disorder, Bipolar Disorder) of 1° relatives* (defined by treatment or diagnosis), if not due to a secondary medical condition.

**Cognitive Domain Calculation**

We calculated five cognitive domains similar to the MATRICS Consensus Cognitive Battery (MCCB) (5,6) and a global cognition score based on individual scores of the cognitive tests that were assessed. The tests used were Diagnostic Analysis of Non-Verbal Accuracy (7), semantic verbal fluency (8), Rey Auditory Verbal Learning Test (9), Trail-Making Test Part A (10), Continuous Performance Test - Identical Pairs (11), Wechsler Memory Scale: spatial span subtest (forward and backward) and the Digit-Symbol-Substitution Task (12). For the social cognition domain we calculated the scaled sum of the correctly identified emotions in the Diagnostic Analysis of Non-Verbal Accuracy (7) test. Working memory was calculated as the scaled average of the correct trials in the backward and forward digit span test (subtest of the Wechsler Adult Intelligence Scale) (12). For the speed of processing domain the scaled number of correctly named words in the semantic verbal fluency task (13) was subtracted from the scaled execution time of the Trail Making Test A (10) and the scaled number of correct digit-symbol substitutions (digit-symbol substitution task) (12) was added. This was then divided by three. We calculated attention as the scaled difference between number of correct answers and the number of errors in the continuous performance test - identical pairs (14). For the verbal learning score the scaled sum of trial 1 to 5 of the Rey Auditory Verbal Learning Task (15) was calculated. Global cognition was calculated as the average of the scaled values across the five domains. Scaling was performed over all subjects including HC. We corrected for confounding effects of age, sex, site and years of education as done before (16). Regression models based on HC were built for every cognition domain using age, sex, site and years of education as predictors. These models were then applied to the patient data and residuals were used for further analysis.

**Correction for site differences via ComBat**

ComBat is a procedure originally developed for correcting batch effects in gene expression data, using an empirical Bayesian framework. Recently, ComBat has been successfully used to account for potentially confounding site-effects in MRI data in over 50 studies (17). We used Munich as a reference site, as it was the site with the highest number of study participants. While correcting for site effects, ComBat allows for specification of variables whose variance should be preserved. To ensure that differences due to biological variation are maintained, we chose to preserve the variance in age and sex. We trained ComBat on HC subjects and the derived estimates were then applied to the patient data using the implementation neuroHarmonize (18).

**Canonical Correlation Analysis**

We used Canonical Correlation Analysis (CCA) (19) to explore if there is a complex connection between gradient metrics and clinical variables. This method identifies variates as linear combinations of gradient metrics that maximally covary with linear combinations of clinical variables. To test statistical significance of the components that represent the relationship between combinations of gradient metrics and combinations of clinical variables we used permutation-based hypothesis testing. Thus, the order of the sample across the features in the original data set was randomized 1000 times. Then CCA was applied to each new randomized dataset, resulting in distributions of correlation values for each component against which the original values were tested. To furthermore investigate the association of the original variables with the components we tested the significance of the loadings, which are the correlations of each feature with a component. We again did this via permutation-based hypothesis testing.

**Supplementary Results**

**Canonical Correlation Analysis**

CCA revealed significant sets of linear combinations between gradient metrics and clinical variables. The first component, with the highest correlation showed a correlation of *r* = .44 (*p*_FDR_ = .030). Components 2 to 7 were also significant (*r* = .28 - .40, *p*_FDR_ = .005 - .047). Across all canonical components only clinical variables loaded significantly on the components. For the first component the strongest loadings were found for verbal learning (*r* = -.51, *p*_FDR_ = .002), GF-R (*r* = .35, *p*_FDR_ = .002), GAF-S (*r* = -.35, *p*_FDR_ = .002), GAF-DI (*r* = -.34, *p*_FDR_ = .002) and speed of processing (*r* = .31, *p*_FDR_ = .002) (Supplementary Figure 17). These overlapped with the variables that were also found to be significantly related to gradient measures in the correlation analyses.

**GLM analysis**

To assess consistency of results, when using an approach where covariates are directly included in the model testing for group effects, we performed GLM analyses: Results remained overall consistent with some differences in FDR significance. For mean gradient network values, all clinical groups showed significantly higher values in the SMN in the visual-to-sensorimotor gradient (ROD: β = 0.41, 95% CI [0.16, 0.66], *p*_FDR_ = .015; ROP: β = 0.45, 95% CI [0.20, 0.69], *p*_FDR_ = .007; CHR-P: β = 0.33, 95% CI [0.09, 0.58], p_FDR_ = .032). Additionally, alterations in the VAN for CHR-P (β = −0.27, 95% CI [−0.45, −0.12], *p*_FDR_ = .020) and in the sensory-to-association gradient for ROP (β = −0.30, 95% CI [−0.47, −0.09], *p*_FDR_ = .022) remained significant after FDR correction.

The significant results for ROD regarding a smaller range (β = -0.95, 95% CI [-1.84, -0.08], *p*_FDR_ = .098) and reduced within-network dispersion (VAN: β = -0.27, 95% CI [-0.48, -0.06], *p*_FDR_ = .108; DAN: β = -0.32, 95% CI [-0.56, -0.07], *p*_FDR_ = .108) did not survive FDR-correction. Regarding the reduced between-network dispersion ROP and CHR-P showed reduced between-network dispersion between the SMN and VAN (ROP: β = -0.56, 95% CI [-0.91, -0.22], *p*_FDR_ = .041; CHR-P: β = -0.63, 95% CI [-0.98, -0.29], *p*_FDR_ = .021) and ROP showed reduced between-network dispersion between the SMN and DAN (β = -0.69, 95% CI [-1.13, -0.25], *p*_FDR_ = .041).

**Supplementary Tables**

**Supplementary Table 1**

| Neuroimaging acquisition parameters for the structural MRI data across the different PRONIA sites. Adapted with minimal modifications from Koutsouleris et al. (2018) (3)/ Buciuman et al. (2023) (20) | | | | | | | | | |
| --- | --- | --- | --- | --- | --- | --- | --- | --- | --- |
| Site | Scanner model | Field strength | Flip Angle | Coil Channels | Voxel size (mm) | TR (ms) | TE (ms) | FOV (mm) | Slices |
| Munich | Philips Ingenia | 3T | 8 | 32 | 0.97 x 0.97 x 1 | 9.5 | 5.5 | 250 x 250 | 190 |
| Milan | Philips Achieva Intera | 1.5T | 12 | 8 | 0.93 x 0.93 x 1 | Shortest (8.1) | Shortest (3.7) | 240 x 240 | 170 |
| Cologne | Philips Achieva | 3T | 8 | 8 | 0.97 x 0.97 x 1 | 9.5 | 5.5 | 250 x 250 | 190 |
| Basel | SIEMENS Verio | 3T | 8 | 12 | 1 x 1 x 1 | 2000 | 3.4 | 256 x 256 | 176 |
| Birming-ham | Philips Achieva | 3T | 8 | 32 | 1 x 1 x 1 | 8.4 | 3.8 | 288 x 287 | 175 |
| Udine | Philips Achieva | 3T | 12 | 8 | 0.93 x 0.93 x 1 | Shortest (8.1) | Shortest (3.7) | 240 x 240 | 170 |
| Münster | SIEMENS Prisma fit | 3T | 8 | 12 | 1 x 1 x 1 | 2130 | 2.3 | 256 x 256 | 192 |
| Turku | Philips Ingenuity | 3T | 7 | 32 | 1 x 1 x 1 | 8.1 | 3.7 | 256 x 256 | 176 |

*Note*. TR = repetition time; TE = echo time; FOV = field of view.

**Supplementary Table 2**

| Neuroimaging acquisition parameters for the resting-state functional MRI data across the different PRONIA sites. Adapted with minimal modifications from Buciuman et al. (2023) | | | | | | | | | |
| --- | --- | --- | --- | --- | --- | --- | --- | --- | --- |
| Site | Scanner model | Field strength | Flip Angle | Coil Channels | Voxel size (mm) | TE (ms) | FOV (mm) | Slices | Slice order |
| Munich | Philips Ingenia | 3T | 90 | 32 | 2.88 x 2.88 x 3 | 30 | 230 x 230 | 53 | Ascending |
| Milan | Philips Achieva Intera | 1.5T | 90 | 8 | 3 x 3 x 3 | 32 | 240 x 240 | 45 | Interleaved |
| Cologne | Philips Achieva | 3T | 90 | 8 | 2.88 x 2.88 x 3 | 30 | 230 x 230 | 53 | Ascending |
| Basel | SIEMENS Verio | 3T | 82 | 12 | 2.98 x 2.98 x 3 | 28 | 256 x 256 | 34 | Interleaved |
| Birming-ham | Philips Achieva | 3T | 85 | 32 | 3 x 3 x 3 | 34.5 | 240 x 240 | 52 | Interleaved |
| Udine | Philips Achieva | 3T | 90 | 8 | 3 x 3 x 3 | 32 | 240 x 240 | 45 | Interleaved |
| Münster | SIEMENS Prisma fit | 3T | 90 | 12 | 3 x 3 x 3 | 26 | 256 x 256 | 51 | Interleaved |
| Turku | Philips Ingenuity | 3T | 90 | 32 | 3 x 3 x 3 | 30 | 240 x 240 | 53 | Interleaved |

*Note*. TR = repetition time; TE = echo time; FOV = field of view.

**Supplementary Table 3**

Receptors included in receptor expression analysis

| Neurotransmitter system | receptors |
| --- | --- |
| acetylcholine | VAChT, (21–23), M1 (24), a4b2 (25) |
| dopamine | D2 (26–30), DAT (31,32), D1 (33) |
| cannabinoid | CB1 (34,35) |
| opioid | KOR (36), MOR (37,38) |
| glutamate | mGlur5 (23,39,40) , NMDA (23) |
| serotonin | 5Ht1a (41,42), 5Ht1b (41–43), 5HTT (41,42,44), 5Ht2a (41,42), 5Ht6 (45), 5Ht4 (42) |
| GABA | GABAa (31,46), GABAa alpha 5 subunit (47) |
| norepinephrene | NET (48,49) |
| histamine | H3 (50) |

**Supplementary Table 4**

Median (IQR) values for gradient metrics by group

| Metric | Network(s)/ gradient | CHR - P | HC | ROD | ROP |
| --- | --- | --- | --- | --- | --- |
| Gradient  1 | CN | -0.06  (-0.43 - 0.35) | 0.07  (-0.39 - 0.51) | 0.03  (-0.45 - 0.34) | -0.03  (-0.45 - 0.43) |
| Gradient  1 | DAN | 0.00  (-0.51 - 0.58) | -0.01  (-0.48 - 0.61) | -0.06  (-0.68 - 0.44) | -0.12  (-0.67 - 0.50) |
| Gradient  1 | DMN | -0.04  (-0.50 - 0.35) | -0.09  (-0.51 - 0.46) | 0.06  (-0.54 - 0.54) | 0.02  (-0.45 - 0.46) |
| Gradient  1 | LN | -0.06  (-0.73 - 0.63) | -0.07  (-0.73 - 0.61) | -0.08  (-0.90 - 0.60) | 0.06  (-0.60 - 0.86) |
| Gradient  1 | SMN | 0.32  (-0.47 - 1.00) | -0.05  (-0.94 - 0.86) | 0.35  (-0.45 - 0.94) | 0.34  (-0.60 - 1.04) |
| Gradient  1 | VAN | -0.01  (-0.60 - 0.61) | 0.31  (-0.38 - 0.82) | 0.09  (-0.48 - 0.68) | -0.03  (-0.72 - 0.57) |
| Gradient  1 | VN | -0.51  (-1.45 - 1.08) | -0.23  (-1.36 - 1.34) | -0.63  (-1.59 - 0.78) | -0.61  (-1.70 - 1.12) |
| Gradient  2 | CN | -0.19  (-0.79 - 0.55) | -0.13  (-0.69 - 0.61) | -0.25  (-0.82 - 0.52) | -0.15  (-0.72 - 0.56) |
| Gradient  2 | DAN | 0.12  (-0.61 - 0.68) | 0.25  (-0.59 - 0.76) | 0.05  (-0.71 - 0.72) | 0.11  (-0.67 - 0.79) |
| Gradient  2 | DMN | -0.42  (-1.24 - 0.80) | -0.20  (-1.19 - 0.92) | -0.14  (-1.04 - 1.01) | -0.13  (-0.98 - 0.88) |
| Gradient 2 | LN | -0.31  (-1.06 - 0.89) | -0.34  (-1.07 - 0.74) | -0.13  (-1.08 - 0.68) | -0.27  (-0.99 - 0.97) |
| Gradient 2 | SMN | 0.09  (-0.61 - 0.95) | 0.15  (-0.84 - 0.86) | 0.11  (-0.58 - 0.76) | 0.12  (-0.73 - 0.86) |
| Gradient 2 | VAN | 0.05  (-0.59 - 0.75) | 0.20  (-0.50 - 0.81) | -0.05  (-0.72 - 0.63) | -0.03  (-0.76 - 0.59) |
| Gradient 2 | VN | 0.23  (-0.77 - 1.06) | 0.09  (-1.11 - 0.95) | 0.30  (-0.76 - 1.11) | 0.35  (-0.84 - 1.12) |
| Range | Gradient 1 | -1.09  (-3.68 - 2.22) | -0.45  (-3.06 - 2.80) | -1.53  (-4.50 - 2.55) | -0.93  (-3.63 - 2.91) |
| Range | Gradient 2 | -0.69  (-3.39 - 2.43) | -0.58  (-2.60 -1.82) | -1.01  (-2.84 - 1.63) | -0.69  (-2.51 - 2.20) |
| WND | CN | -0.25  (-0.90 - 0.77) | -0.35  (-0.95 - 0.42) | -0.23  (-0.87 - 0.48) | -0.10  (-0.74 - 0.70) |
| WND | DAN | -0.32  (-1.04 - 0.62) | -0.16  (-0.81 - 0.79) | -0.51  (-1.15 - 0.57) | -0.25  (-0.80 - 0.72) |
| WND | DMN | -0.31  (-0.85 - 0.44) | -0.37  (-0.88 - 0.51) | -0.36  (-0.90 - 0.49) | -0.22  (-0.74 - 0.69) |
| WND | LN | -0.42  (-1.08 - 0.65) | -0.36  (-1.01 - 0.76) | -0.32  (-1.03 - 0.56) | -0.44  (-1.09 - 0.73) |
| WND | SMN | -0.27  (-0.76 - 0.37) | -0.24  (-0.78 - 0.42) | -0.20  (-0.82 - 0.34) | -0.04  (-0.64 - 0.67) |
| WND | VAN | -0.17  (-0.85 - 0.54) | -0.14  (-0.70 - 0.61) | -0.42  (-0.84 - 0.17) | -0.28  (-0.76 - 0.33) |
| WND | VN | -0.35  (-0.87 - 0.42) | -0.21  (-0.83 - 0.79) | -0.34  (-1.00 - 0.36) | -0.37  (-0.96 - 0.58) |
| BND | DMN - CN | -0.49  (-1.49 - 0.88) | -0.49  (-1.43 - 0.93) | -0.25  (-1.19 - 1.24) | -0.15  (-1.15 - 1.19) |
| BND | DMN - DAN | -0.59  (-2.23 -. 1.86) | -0.69  (-2.50 - 1.45) | -0.02  (-2.13 - 2.25) | -0.06  (-1.74 - 1.77) |
| BND | DMN - VAN | -0.74  (-2.34 - 1.83) | -0.73  (-2.46 - 1.31) | -0.37  (-2.21 - 2.15) | -0.05  (-1.97 - 2.03) |
| BND | DAN - CN | -0.31  (-1.43 - 1.18) | -0.56  (-1.55 - 0.75) | -0.48  (-1.47 - 0.77) | -0.43  (-1.53 - 1.17) |
| BND | DAN - VAN | -0.24  (-1.05 - 0.72) | -0.47  (-1.24 - 0.63) | -0.56  (-1.20 - 0.89) | -0.48  (-1.24 - 1.08) |
| BND | LN - CN | -0.85  (-2.16 - 0.93) | -1.03  (-2.29 - 1.27) | -0.69  (-2.02 - 1.02) | -0.57  (-2.23 - 1.32) |
| BND | LN - DMN | -0.98  (-2.24 - 1.09) | -0.66  (-2.19 - 1.62) | -0.47  (-1.98 - 1.39) | -1.21  (-2.28 - 0.79) |
| BND | LN - DAN | -0.92  (-2.95 - 2.04) | -1.06  (-3.10 - 1.82) | -0.56  (-2.74 - 1.59) | -0.37  (-2.42 - 2.34) |
| BND | LN - VAN | -0.98  (-2.60 - 1.93) | -0.93  (-2.86 - 1.65) | -0.93  (-2.87 - 1.78) | -0.52  (-2.34 - 2.51) |
| BND | LN - SMN | -1.07  (-3.24 - 2.45) | -0.72  (-2.89 - 2.37) | -0.98  (-3.64 - 1.40) | -0.65  (-2.98 - 2.42) |
| BND | LN - VN | -0.99  (-3.43 - 2.32) | -0.54  (-2.85 - 3.03) | -0.80  (-3.33 - 1.82) | -0.54  (-3.19 - 2.56) |
| BND | VAN - CN | -0.48  (-1.75 - 0.82) | -0.65  (-1.74 - 0.87) | -0.60  (-1.83 - 1.19) | -0.44  (-1.64 - 1.46) |
| BND | SMN - CN | -0.80  (-2.53 - 1.61) | -0.64  (-2.15 - 2.31) | -1.28  (-2.58 - 1.44) | -0.91  (-2.32 - 1.57) |
| BND | SMN - DMN | -0.83  (-2.65 - 1.53) | -0.41  (-2.60 - 1.96) | -0.85  (-2.64 - 2.05) | -0.32  (-2.35 - 1.98) |
| BND | SMN - DAN | -0.57  (-1.74 - 0.80) | -0.38  (-1.51 - 1.76) | -1.06  (-2.04 - 0.96) | -0.82  (-1.99 - 0.73) |
| BND | SMN - VAN | -0.80  (-1.34 - 0.34) | -0.42  (-1.23 - 1.10) | -0.73  (-1.31 - 0.42) | -0.70  (-1.26 - 0.10) |
| BND | VN - CN | -0.66  (-2.77 - 1.36) | -0.43  (-2.24 - 2.28) | -0.71  (-2.97 - 1.58) | -0.67  (-2.54 - 2.26) |
| BND | VN - DMN | -0.74  (-2.82–1.93) | -0.23  (-2.40–2.20) | -0.56  (-2.84–2.07) | -0.35  (-2.45–2.25) |
| BND | VN - DAN | -1.00  (-2.45 - 1.15) | -0.47  (-2.01 - 1.77) | -0.98  (-2.37 - 1.29) | -0.87  (-2.39 - 1.45) |
| BND | VN - VAN | -0.64  (-2.67 - 2.05) | -0.78  (-2.82 - 2.07) | -1.01  (-3.03 - 1.59) | -0.56  (-2.79 - 2.50) |
| BND | VN - SMN | -0.97  (-2.98 - 1.73) | -0.34  (-2.80 - 2.83) | -1.46  (-3.27 - 1.48) | -1.01  (-3.25 - 1.99) |

*Note.* BND = between-network dispersion; CHR-P = clinical high-risk; CN = control network; DAN = dorsal attention network; DMN = default-mode network; HC = healthy control; LN = limbic network; ROD = recent-onset depression; ROP = recent-onset psychosis; SMN = somatomotor network; VAN = ventral attention network; VN = visual network; WND = within-network dispersion.

**Supplementary Table 5**

FDR corrected p-values for correlations between within-network dispersion and clinical variables

| Clinical Variable | LN | CN | DAN | VAN | VN | DMN | SMN |
| --- | --- | --- | --- | --- | --- | --- | --- |
| Cog total | 0.693 | 0.693 | 0.320 | 0.503 | 0.401 | 0.622 | 0.049 |
| WM | 0.929 | 0.945 | 0.663 | 0.748 | 0.622 | 0.929 | 0.320 |
| Soc Cog | 0.320 | 0.401 | 0.320 | 0.426 | 0.320 | 0.624 | 0.624 |
| Attn | 1.000 | 0.693 | 0.891 | 0.945 | 0.726 | 1.000 | 0.320 |
| VerLrn | 0.624 | 0.301 | 0.301 | 0.320 | 0.929 | 0.320 | 0.049 |
| SoP | 0.566 | 0.693 | 0.320 | 0.406 | 0.320 | 0.696 | 0.301 |
| BDI | 0.693 | 0.693 | 0.473 | 0.320 | 0.693 | 0.945 | 0.320 |
| GAF S | 0.693 | 0.473 | 0.406 | 0.997 | 0.406 | 0.945 | 0.473 |
| GAF DI | 0.748 | 0.401 | 0.929 | 0.693 | 0.406 | 0.693 | 0.562 |
| GF S | 0.706 | 0.566 | 0.929 | 0.693 | 0.606 | 0.624 | 0.427 |
| GF R | 0.610 | 0.301 | 0.885 | 0.885 | 0.320 | 0.693 | 0.939 |
| PANSS Positive | 0.988 | 0.696 | 0.126 | 0.406 | 0.728 | 0.668 | 0.073 |
| PANSS Negative | 0.696 | 0.438 | 0.320 | 0.851 | 0.320 | 0.693 | 0.693 |
| PANSS General | 0.434 | 0.726 | 0.406 | 0.945 | 0.320 | 0.885 | 0.426 |
| VisDys | 0.668 | 0.301 | 0.049 | 0.624 | 0.320 | 0.434 | 0.406 |

*Note.* Attn = attention; BDI = Becks Depression Inventory; CN = control network; Cog total = cognition total score; DAN = dorsal attention network; DMN = default-mode network; GAF DI = Global Assessment of Functioning Disability; GAF S = Global Assessment of Functioning Symptoms; GF R = Global Functioning Role Scale; GF S = Global Functioning Social Scale; LN = limbic network; Soc Cog = social cognition; SoP = speed of processing; SMN = somatomotor network; VAN = ventral attention network; VerLrn = verbal learning; VisDys = visual dysfunctions; VN = visual network; WM = working memory.

**Supplementary Table 6**

FDR corrected p-values for correlations between between-network dispersion and clinical variables

| Clinical Variable | Network | FDR pvalue |
| --- | --- | --- |
| Cog total | LN-VN | 0.934 |
| WM | LN-VN | 0.978 |
| Soc Cog | LN-VN | 0.989 |
| Attn | LN-VN | 0.937 |
| VerLrn | LN-VN | 0.989 |
| SoP | LN-VN | 0.881 |
| BDI | LN-VN | 0.881 |
| GAF S | LN-VN | 0.979 |
| GAF DI | LN-VN | 0.978 |
| GF S | LN-VN | 0.978 |
| GF R | LN-VN | 0.978 |
| PANSS Positive | LN-VN | 0.786 |
| PANSS Negative | LN-VN | 0.514 |
| PANSS General | LN-VN | 0.619 |
| VisDys | LN-VN | 0.514 |
| Cog total | LN-SMN | 0.981 |
| WM | LN-SMN | 0.973 |
| Soc Cog | LN-SMN | 0.934 |
| Attn | LN-SMN | 0.988 |
| VerLrn | LN-SMN | 0.988 |
| SoP | LN-SMN | 0.978 |
| BDI | LN-SMN | 0.978 |
| GAF S | LN-SMN | 0.851 |
| GAF DI | LN-SMN | 0.769 |
| GF S | LN-SMN | 0.981 |
| GF R | LN-SMN | 0.934 |
| PANSS Positive | LN-SMN | 0.605 |
| PANSS Negative | LN-SMN | 0.514 |
| PANSS General | LN-SMN | 0.516 |
| VisDys | LN-SMN | 0.514 |
| Cog total | LN-DMN | 0.977 |
| WM | LN-DMN | 0.779 |
| Soc Cog | LN-DMN | 0.769 |
| Attn | LN-DMN | 0.934 |
| VerLrn | LN-DMN | 0.988 |
| SoP | LN-DMN | 0.978 |
| BDI | LN-DMN | 0.978 |
| GAF S | LN-DMN | 0.988 |
| GAF DI | LN-DMN | 0.934 |
| GF S | LN-DMN | 0.569 |
| GF R | LN-DMN | 0.946 |
| PANSS Positive | LN-DMN | 0.514 |
| PANSS Negative | LN-DMN | 0.988 |
| PANSS General | LN-DMN | 0.978 |
| VisDys | LN-DMN | 0.973 |
| Cog total | LN-DAN | 0.988 |
| WM | LN-DAN | 0.946 |
| Soc Cog | LN-DAN | 0.940 |
| Attn | LN-DAN | 0.973 |
| VerLrn | LN-DAN | 0.978 |
| SoP | LN-DAN | 0.978 |
| BDI | LN-DAN | 0.978 |
| GAF S | LN-DAN | 0.786 |
| GAF DI | LN-DAN | 0.514 |
| GF S | LN-DAN | 0.516 |
| GF R | LN-DAN | 0.516 |
| PANSS Positive | LN-DAN | 0.654 |
| PANSS Negative | LN-DAN | 0.514 |
| PANSS General | LN-DAN | 0.769 |
| VisDys | LN-DAN | 0.240 |
| Cog total | LN-VAN | 0.978 |
| WM | LN-VAN | 0.978 |
| Soc Cog | LN-VAN | 0.934 |
| Attn | LN-VAN | 0.973 |
| VerLrn | LN-VAN | 0.981 |
| SoP | LN-VAN | 0.973 |
| BDI | LN-VAN | 0.934 |
| GAF S | LN-VAN | 0.769 |
| GAF DI | LN-VAN | 0.536 |
| GF S | LN-VAN | 0.786 |
| GF R | LN-VAN | 0.634 |
| PANSS Positive | LN-VAN | 0.525 |
| PANSS Negative | LN-VAN | 0.514 |
| PANSS General | LN-VAN | 0.514 |
| VisDys | LN-VAN | 0.514 |
| Cog total | LN-CN | 0.978 |
| WM | LN-CN | 0.642 |
| Soc Cog | LN-CN | 0.610 |
| Attn | LN-CN | 0.914 |
| VerLrn | LN-CN | 0.973 |
| SoP | LN-CN | 0.786 |
| BDI | LN-CN | 0.988 |
| GAF S | LN-CN | 0.973 |
| GAF DI | LN-CN | 0.978 |
| GF S | LN-CN | 0.973 |
| GF R | LN-CN | 0.978 |
| PANSS Positive | LN-CN | 0.981 |
| PANSS Negative | LN-CN | 0.981 |
| PANSS General | LN-CN | 0.946 |
| VisDys | LN-CN | 0.973 |
| Cog total | VN-SMN | 0.851 |
| WM | VN-SMN | 0.769 |
| Soc Cog | VN-SMN | 0.516 |
| Attn | VN-SMN | 0.946 |
| VerLrn | VN-SMN | 0.988 |
| SoP | VN-SMN | 0.837 |
| BDI | VN-SMN | 0.988 |
| GAF S | VN-SMN | 0.851 |
| GAF DI | VN-SMN | 0.516 |
| GF S | VN-SMN | 0.514 |
| GF R | VN-SMN | 0.569 |
| PANSS Positive | VN-SMN | 0.619 |
| PANSS Negative | VN-SMN | 0.978 |
| PANSS General | VN-SMN | 0.978 |
| VisDys | VN-SMN | 0.295 |
| Cog total | VN-DMN | 0.988 |
| WM | VN-DMN | 0.978 |
| Soc Cog | VN-DMN | 0.514 |
| Attn | VN-DMN | 0.988 |
| VerLrn | VN-DMN | 0.978 |
| SoP | VN-DMN | 0.919 |
| BDI | VN-DMN | 0.851 |
| GAF S | VN-DMN | 0.978 |
| GAF DI | VN-DMN | 0.934 |
| GF S | VN-DMN | 0.940 |
| GF R | VN-DMN | 0.978 |
| PANSS Positive | VN-DMN | 0.973 |
| PANSS Negative | VN-DMN | 0.786 |
| PANSS General | VN-DMN | 0.678 |
| VisDys | VN-DMN | 0.639 |
| Cog total | VN-DAN | 0.667 |
| WM | VN-DAN | 0.516 |
| Soc Cog | VN-DAN | 0.516 |
| Attn | VN-DAN | 0.891 |
| VerLrn | VN-DAN | 0.851 |
| SoP | VN-DAN | 0.851 |
| BDI | VN-DAN | 0.946 |
| GAF S | VN-DAN | 0.978 |
| GAF DI | VN-DAN | 0.981 |
| GF S | VN-DAN | 0.861 |
| GF R | VN-DAN | 0.694 |
| PANSS Positive | VN-DAN | 0.684 |
| PANSS Negative | VN-DAN | 0.988 |
| PANSS General | VN-DAN | 0.851 |
| VisDys | VN-DAN | 0.514 |
| Cog total | VN-VAN | 0.516 |
| WM | VN-VAN | 0.517 |
| Soc Cog | VN-VAN | 0.514 |
| Attn | VN-VAN | 0.851 |
| VerLrn | VN-VAN | 0.937 |
| SoP | VN-VAN | 0.628 |
| BDI | VN-VAN | 0.934 |
| GAF S | VN-VAN | 0.976 |
| GAF DI | VN-VAN | 0.628 |
| GF S | VN-VAN | 0.514 |
| GF R | VN-VAN | 0.573 |
| PANSS Positive | VN-VAN | 0.516 |
| PANSS Negative | VN-VAN | 0.988 |
| PANSS General | VN-VAN | 0.934 |
| VisDys | VN-VAN | 0.240 |
| Cog total | VN-CN | 0.786 |
| WM | VN-CN | 0.881 |
| Soc Cog | VN-CN | 0.514 |
| Attn | VN-CN | 0.989 |
| VerLrn | VN-CN | 0.881 |
| SoP | VN-CN | 0.779 |
| BDI | VN-CN | 0.978 |
| GAF S | VN-CN | 0.978 |
| GAF DI | VN-CN | 0.934 |
| GF S | VN-CN | 0.725 |
| GF R | VN-CN | 0.769 |
| PANSS Positive | VN-CN | 0.851 |
| PANSS Negative | VN-CN | 0.690 |
| PANSS General | VN-CN | 0.934 |
| VisDys | VN-CN | 0.765 |
| Cog total | SMN-DMN | 0.769 |
| WM | SMN-DMN | 0.536 |
| Soc Cog | SMN-DMN | 0.516 |
| Attn | SMN-DMN | 0.769 |
| VerLrn | SMN-DMN | 0.875 |
| SoP | SMN-DMN | 0.978 |
| BDI | SMN-DMN | 0.978 |
| GAF S | SMN-DMN | 0.991 |
| GAF DI | SMN-DMN | 0.724 |
| GF S | SMN-DMN | 0.797 |
| GF R | SMN-DMN | 0.769 |
| PANSS Positive | SMN-DMN | 0.973 |
| PANSS Negative | SMN-DMN | 0.514 |
| PANSS General | SMN-DMN | 0.516 |
| VisDys | SMN-DMN | 0.569 |
| Cog total | SMN-DAN | 0.654 |
| WM | SMN-DAN | 0.653 |
| Soc Cog | SMN-DAN | 0.628 |
| Attn | SMN-DAN | 0.881 |
| VerLrn | SMN-DAN | 0.514 |
| SoP | SMN-DAN | 0.934 |
| BDI | SMN-DAN | 0.978 |
| GAF S | SMN-DAN | 0.514 |
| GAF DI | SMN-DAN | 0.223 |
| GF S | SMN-DAN | 0.223 |
| GF R | SMN-DAN | 0.482 |
| PANSS Positive | SMN-DAN | 0.639 |
| PANSS Negative | SMN-DAN | 0.978 |
| PANSS General | SMN-DAN | 0.934 |
| VisDys | SMN-DAN | 0.634 |
| Cog total | SMN-VAN | 0.514 |
| WM | SMN-VAN | 0.583 |
| Soc Cog | SMN-VAN | 0.881 |
| Attn | SMN-VAN | 0.514 |
| VerLrn | SMN-VAN | 0.514 |
| SoP | SMN-VAN | 0.861 |
| BDI | SMN-VAN | 0.724 |
| GAF S | SMN-VAN | 0.516 |
| GAF DI | SMN-VAN | 0.634 |
| GF S | SMN-VAN | 0.654 |
| GF R | SMN-VAN | 0.516 |
| PANSS Positive | SMN-VAN | 0.981 |
| PANSS Negative | SMN-VAN | 0.747 |
| PANSS General | SMN-VAN | 0.988 |
| VisDys | SMN-VAN | 0.769 |
| Cog total | SMN-CN | 0.881 |
| WM | SMN-CN | 0.516 |
| Soc Cog | SMN-CN | 0.684 |
| Attn | SMN-CN | 0.626 |
| VerLrn | SMN-CN | 0.973 |
| SoP | SMN-CN | 0.974 |
| BDI | SMN-CN | 0.984 |
| GAF S | SMN-CN | 0.973 |
| GAF DI | SMN-CN | 0.946 |
| GF S | SMN-CN | 0.988 |
| GF R | SMN-CN | 0.978 |
| PANSS Positive | SMN-CN | 0.978 |
| PANSS Negative | SMN-CN | 0.240 |
| PANSS General | SMN-CN | 0.514 |
| VisDys | SMN-CN | 0.973 |
| Cog total | DMN-DAN | 0.797 |
| WM | DMN-DAN | 0.769 |
| Soc Cog | DMN-DAN | 0.891 |
| Attn | DMN-DAN | 0.851 |
| VerLrn | DMN-DAN | 0.934 |
| SoP | DMN-DAN | 0.988 |
| BDI | DMN-DAN | 0.851 |
| GAF S | DMN-DAN | 0.978 |
| GAF DI | DMN-DAN | 0.514 |
| GF S | DMN-DAN | 0.569 |
| GF R | DMN-DAN | 0.514 |
| PANSS Positive | DMN-DAN | 0.881 |
| PANSS Negative | DMN-DAN | 0.642 |
| PANSS General | DMN-DAN | 0.881 |
| VisDys | DMN-DAN | 0.514 |
| Cog total | DMN-VAN | 0.934 |
| WM | DMN-VAN | 0.851 |
| Soc Cog | DMN-VAN | 0.804 |
| Attn | DMN-VAN | 0.786 |
| VerLrn | DMN-VAN | 0.973 |
| SoP | DMN-VAN | 0.934 |
| BDI | DMN-VAN | 0.628 |
| GAF S | DMN-VAN | 0.797 |
| GAF DI | DMN-VAN | 0.443 |
| GF S | DMN-VAN | 0.516 |
| GF R | DMN-VAN | 0.295 |
| PANSS Positive | DMN-VAN | 0.981 |
| PANSS Negative | DMN-VAN | 0.514 |
| PANSS General | DMN-VAN | 0.514 |
| VisDys | DMN-VAN | 0.514 |
| Cog total | DMN-CN | 0.934 |
| WM | DMN-CN | 0.871 |
| Soc Cog | DMN-CN | 0.988 |
| Attn | DMN-CN | 0.934 |
| VerLrn | DMN-CN | 0.988 |
| SoP | DMN-CN | 0.516 |
| BDI | DMN-CN | 0.831 |
| GAF S | DMN-CN | 0.988 |
| GAF DI | DMN-CN | 0.569 |
| GF S | DMN-CN | 0.516 |
| GF R | DMN-CN | 0.626 |
| PANSS Positive | DMN-CN | 0.973 |
| PANSS Negative | DMN-CN | 0.991 |
| PANSS General | DMN-CN | 0.939 |
| VisDys | DMN-CN | 0.514 |
| Cog total | DAN-VAN | 0.862 |
| WM | DAN-VAN | 0.861 |
| Soc Cog | DAN-VAN | 0.593 |
| Attn | DAN-VAN | 0.789 |
| VerLrn | DAN-VAN | 0.514 |
| SoP | DAN-VAN | 0.915 |
| BDI | DAN-VAN | 0.981 |
| GAF S | DAN-VAN | 0.934 |
| GAF DI | DAN-VAN | 0.569 |
| GF S | DAN-VAN | 0.223 |
| GF R | DAN-VAN | 0.851 |
| PANSS Positive | DAN-VAN | 0.569 |
| PANSS Negative | DAN-VAN | 0.981 |
| PANSS General | DAN-VAN | 0.978 |
| VisDys | DAN-VAN | 0.482 |
| Cog total | DAN-CN | 0.934 |
| WM | DAN-CN | 0.786 |
| Soc Cog | DAN-CN | 0.891 |
| Attn | DAN-CN | 0.786 |
| VerLrn | DAN-CN | 0.891 |
| SoP | DAN-CN | 0.934 |
| BDI | DAN-CN | 0.851 |
| GAF S | DAN-CN | 0.973 |
| GAF DI | DAN-CN | 0.851 |
| GF S | DAN-CN | 0.936 |
| GF R | DAN-CN | 0.851 |
| PANSS Positive | DAN-CN | 0.786 |
| PANSS Negative | DAN-CN | 0.514 |
| PANSS General | DAN-CN | 0.984 |
| VisDys | DAN-CN | 0.723 |
| Cog total | VAN-CN | 0.934 |
| WM | VAN-CN | 0.628 |
| Soc Cog | VAN-CN | 0.725 |
| Attn | VAN-CN | 0.569 |
| VerLrn | VAN-CN | 0.926 |
| SoP | VAN-CN | 0.881 |
| BDI | VAN-CN | 0.634 |
| GAF S | VAN-CN | 0.709 |
| GAF DI | VAN-CN | 0.569 |
| GF S | VAN-CN | 0.851 |
| GF R | VAN-CN | 0.514 |
| PANSS Positive | VAN-CN | 0.898 |
| PANSS Negative | VAN-CN | 0.223 |
| PANSS General | VAN-CN | 0.394 |
| VisDys | VAN-CN | 0.973 |

*Note.* Attn = attention; BDI = Becks Depression Inventory; CN = control network; Cog total = cognition total score; DAN = dorsal attention network; DMN = default-mode network; GAF DI = Global Assessment of Functioning Disability; GAF S = Global Assessment of Functioning Symptoms; GF R = Global Functioning Role Scale; GF S = Global Functioning Social Scale; LN = limbic network; Soc Cog = social cognition; SoP = speed of processing; SMN = somatomotor network; VAN = ventral attention network; VerLrn = verbal learning; VisDys = visual dysfunctions; VN = visual network; WM = working memory.

**Supplementary Table 7**

FDR corrected p-values for correlations between mean network values of gradient 1 and clinical variables

| Clinical Variable | LN | CN | DAN | VAN | VN | DMN | SMN |
| --- | --- | --- | --- | --- | --- | --- | --- |
| Cog total | 0.484 | 0.231 | 0.538 | 0.433 | 0.538 | 0.550 | 0.978 |
| WM | 0.538 | 0.215 | 0.724 | 0.782 | 0.538 | 0.484 | 0.725 |
| Soc Cog | 0.132 | 0.807 | 0.737 | 0.081 | 0.231 | 0.322 | 0.124 |
| Attn | 0.872 | 0.601 | 0.673 | 0.681 | 0.538 | 0.433 | 0.988 |
| VerLrn | 0.978 | 0.625 | 0.484 | 0.758 | 0.978 | 0.484 | 0.538 |
| SoP | 0.215 | 0.282 | 0.930 | 0.150 | 0.758 | 0.775 | 0.660 |
| BDI | 0.143 | 0.606 | 0.823 | 0.944 | 0.836 | 0.682 | 0.978 |
| GAF S | 0.601 | 0.969 | 0.124 | 0.614 | 0.553 | 0.561 | 0.693 |
| GAF DI | 0.348 | 0.682 | 0.030 | 0.978 | 0.189 | 0.132 | 0.433 |
| GF S | 0.737 | 0.660 | 0.030 | 0.538 | 0.132 | 0.215 | 0.348 |
| GF R | 0.538 | 0.538 | 0.127 | 0.836 | 0.243 | 0.124 | 0.660 |
| PANSS Positive | 0.693 | 0.601 | 0.737 | 0.132 | 0.569 | 0.981 | 0.540 |
| PANSS Negative | 0.282 | 0.124 | 0.433 | 0.538 | 0.999 | 0.132 | 0.782 |
| PANSS General | 0.737 | 0.348 | 0.538 | 0.298 | 0.978 | 0.282 | 0.693 |
| VisDys | 0.875 | 0.693 | 0.681 | 0.030 | 0.079 | 0.636 | 0.205 |

*Note.* Attn = attention; BDI = Becks Depression Inventory; CN = control network; Cog total = cognition total score; DAN = dorsal attention network; DMN = default-mode network; GAF DI = Global Assessment of Functioning Disability; GAF S = Global Assessment of Functioning Symptoms; GF R = Global Functioning Role Scale; GF S = Global Functioning Social Scale; LN = limbic network; Soc Cog = social cognition; SoP = speed of processing; SMN = somatomotor network; VAN = ventral attention network; VerLrn = verbal learning; VisDys = visual dysfunctions; VN = visual network; WM = working memory.

**Supplementary Table 8**

FDR corrected p-values for correlations between mean network values of gradient 2 and clinical variables

| Clinical Variable | LN | CN | DAN | VAN | VN | DMN | SMN |
| --- | --- | --- | --- | --- | --- | --- | --- |
| Cog total | 0.463 | 0.965 | 0.632 | 0.705 | 0.805 | 0.463 | 0.433 |
| WM | 0.571 | 0.805 | 0.860 | 0.463 | 0.899 | 0.485 | 0.433 |
| Soc Cog | 0.895 | 0.463 | 0.907 | 0.724 | 0.907 | 0.485 | 0.433 |
| Attn | 0.895 | 0.632 | 0.632 | 0.607 | 0.992 | 0.463 | 0.433 |
| VerLrn | 0.792 | 0.776 | 0.899 | 0.952 | 0.907 | 0.697 | 0.451 |
| SoP | 0.433 | 0.965 | 0.805 | 0.965 | 0.805 | 0.907 | 0.965 |
| BDI | 0.761 | 0.907 | 0.792 | 0.433 | 0.705 | 0.788 | 0.935 |
| GAF S | 0.788 | 0.907 | 0.965 | 0.433 | 0.652 | 0.935 | 0.907 |
| GAF DI | 0.632 | 0.968 | 0.547 | 0.018 | 0.792 | 0.433 | 0.632 |
| GF S | 0.895 | 0.935 | 0.463 | 0.057 | 0.632 | 0.433 | 0.792 |
| GF R | 0.697 | 0.899 | 0.451 | 0.018 | 0.632 | 0.451 | 0.632 |
| PANSS Positive | 0.632 | 0.975 | 0.632 | 0.935 | 0.965 | 0.805 | 0.935 |
| PANSS Negative | 0.433 | 0.463 | 0.792 | 0.433 | 0.792 | 0.463 | 0.433 |
| PANSS General | 0.490 | 0.652 | 0.776 | 0.433 | 0.788 | 0.632 | 0.463 |
| VisDys | 0.433 | 0.907 | 0.463 | 0.717 | 0.571 | 0.632 | 0.965 |

*Note.* Attn = attention; BDI = Becks Depression Inventory; CN = control network; Cog total = cognition total score; DAN = dorsal attention network; DMN = default-mode network; GAF DI = Global Assessment of Functioning Disability; GAF S = Global Assessment of Functioning Symptoms; GF R = Global Functioning Role Scale; GF S = Global Functioning Social Scale; LN = limbic network; Soc Cog = social cognition; SoP = speed of processing; SMN = somatomotor network; VAN = ventral attention network; VerLrn = verbal learning; VisDys = visual dysfunctions; VN = visual network; WM = working memory.

**Supplementary Table 9**

FDR corrected p-values for correlations between receptor maps and gradient t-maps

| Receptor | CHR-P Gradient 1 | ROD Gradient 1 | ROP Gradient 1 | CHR-P Gradient 2 | ROD Gradient 2 | ROP Gradient 2 |
| --- | --- | --- | --- | --- | --- | --- |
| mean DAT | 0.240 | 0.612 | 0.850 | **0.041** | 0.441 | 0.242 |
| D1 | 0.240 | 0.517 | 0.850 | **0.022** | 0.169 | 0.447 |
| Mean D2 | 0.322 | 0.612 | 0.964 | **0.019** | **0.020** | 0.322 |
| Mean CB1 | 0.348 | 0.634 | 0.850 | **0.039** | **0.014** | 0.418 |
| mean MOR | 0.322 | 0.818 | 0.951 | **0.031** | **0.017** | 0.365 |
| KOR | 0.783 | 0.845 | 0.850 | 0.818 | 0.731 | 0.365 |
| Mean Vacht | 0.643 | 0.818 | 0.850 | 0.467 | 0.830 | 0.242 |
| M1 | 0.699 | 0.485 | 0.850 | **0.034** | **0.002** | 0.479 |
| a4b2 | 0.913 | 0.818 | 0.951 | **0.033** | 0.441 | 0.365 |
| Mean mGluR5 | 0.215 | 0.456 | 0.850 | **0.017** | **0.009** | 0.242 |
| nmda | 0.699 | 0.818 | 0.964 | 0.098 | 0.441 | 0.322 |
| Mean 5Ht1a | 0.346 | 0.634 | 0.951 | **0.045** | 0.076 | 0.592 |
| Mean 5Ht1b | 0.322 | 0.634 | 0.106 | 0.428 | 0.116 | 0.418 |
| Mean 5HTT | 0.348 | 0.797 | 0.951 | 0.818 | 0.441 | 0.445 |
| Mean 5Ht2a | 0.215 | 0.456 | 0.355 | **0.019** | **0.007** | 0.552 |
| Serotonin 5Ht6 | 0.370 | 0.605 | 0.850 | **0.022** | 0.065 | 0.365 |
| Serotonin 5Ht4 | 0.288 | 0.456 | 0.850 | **0.022** | **0.014** | 0.592 |
| meanGABAa | 0.322 | 0.456 | 0.355 | 0.325 | 0.441 | 0.757 |
| GABAa alpha5 | 0.215 | 0.612 | 0.850 | **0.045** | 0.190 | 0.592 |
| Mean NET | 0.178 | **0.031** | 0.106 | 0.552 | 0.301 | 0.242 |
| pvalH3_fdr | 0.322 | 0.869 | 0.85 | 0.282 | 0.612 | 0.365 |

*Note.* CHR-P = clinical high-risk**;** ROD = recent-onset depression; ROP = recent-onset psychosis.

**Supplementary Figures**


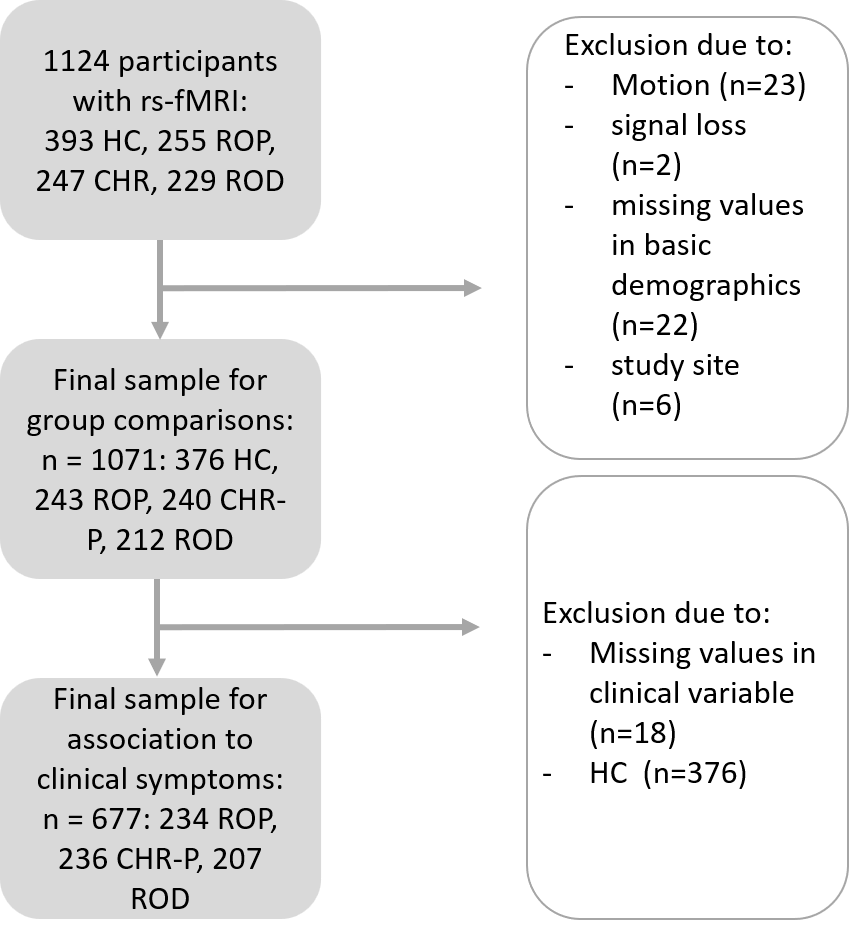


**Supplementary Figure 1.** Flow chart of exclusion of cases.


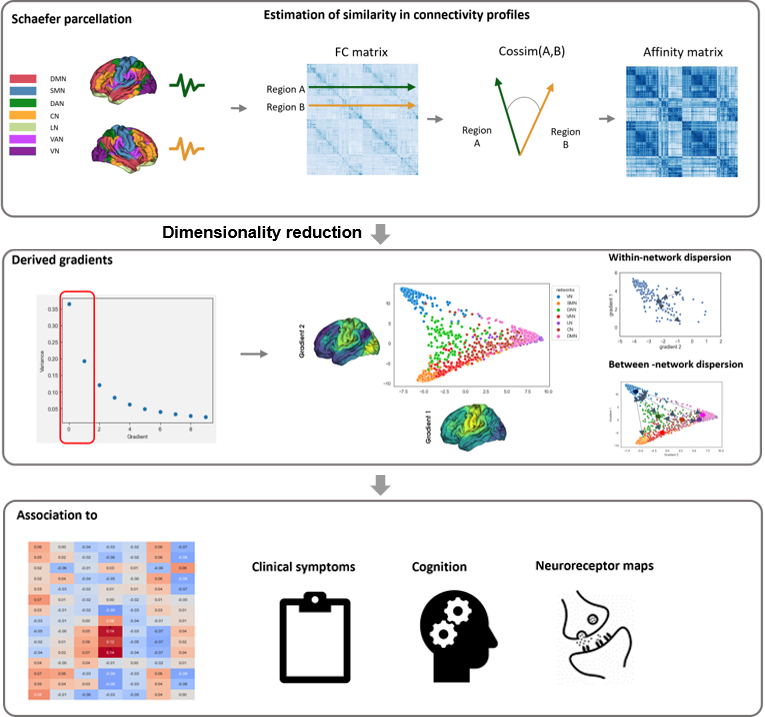


**Supplementary Figure 2.** Analysis pipeline. CN = control network; Cossim = Cosine similarity; DAN = dorsal attention network; DMN = default mode network; FC = functional connectivity; LN = limbic network; SMN = somatomotor network; VAN = ventral attention network; VN = visual network.


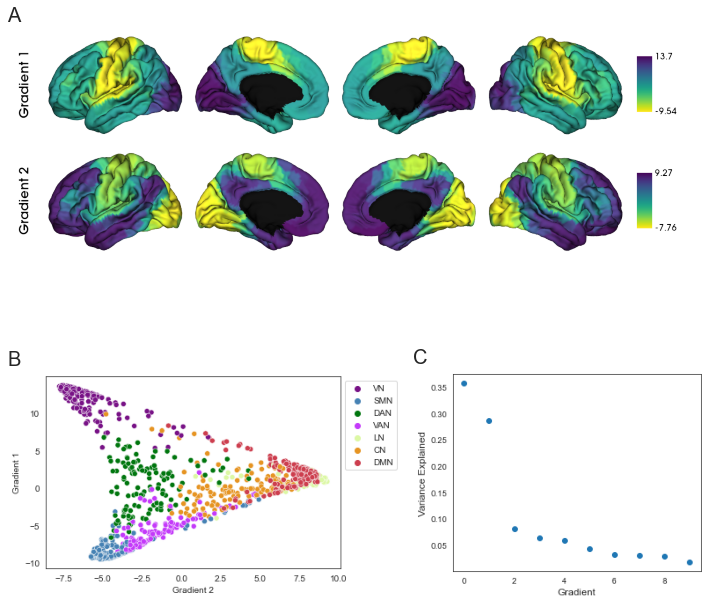


**Supplementary Figure 3.** Reference gradients derived from mean FC of healthy subjects. (A) Spatial topography of first and second gradient. (B) Gradient scores in the two-dimensional gradient space. (C) Variance explained by gradients. VN = visual network; SMN = somatomotor network; DAN = dorsal attention network; VAN = salience ventral attention network; LN = limbic network; CN = control network; DMN = default mode network.


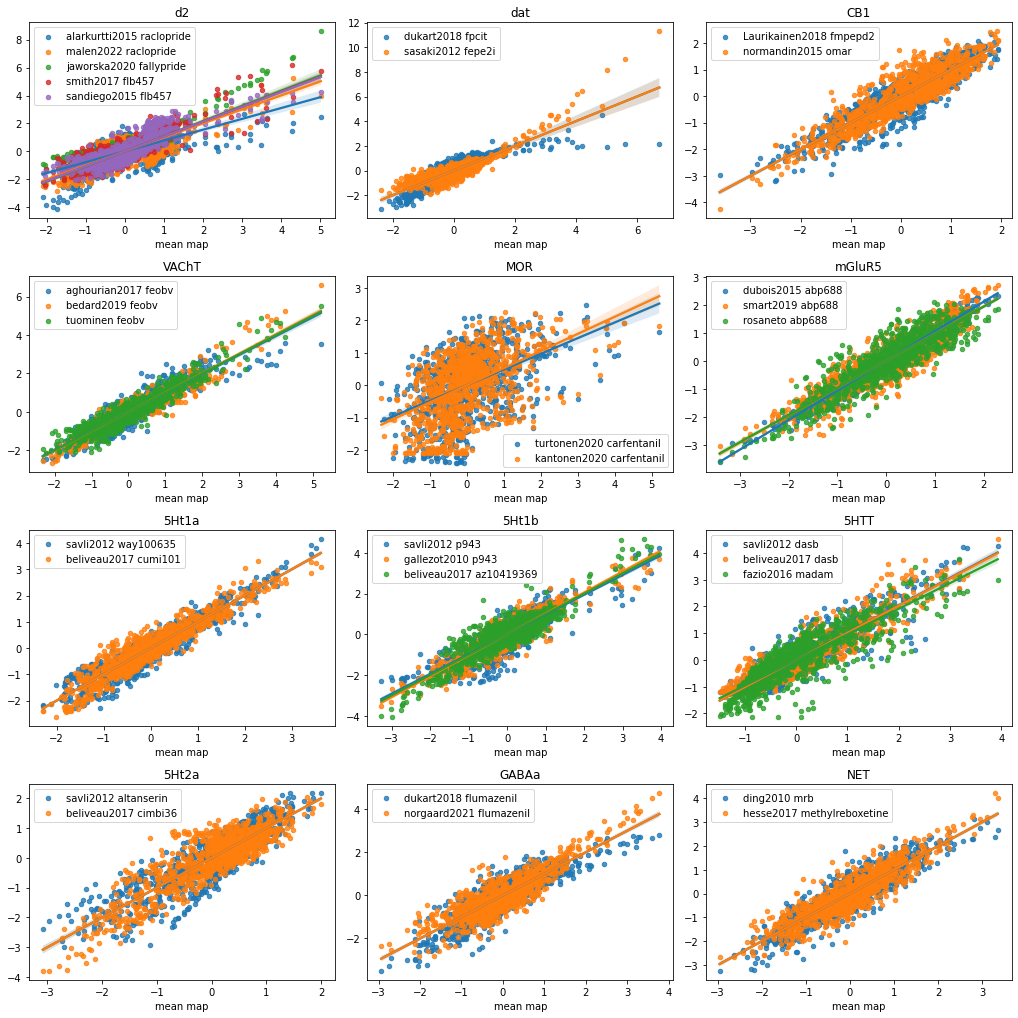


**Supplementary Figure 4.** Correlations of mean receptor maps with single datasets. Maps of the same neurotransmitter were averaged into a single mean neurotransmitter map. Each map is highly correlated with the mean map. Names indicate the dataset and the used tracer.


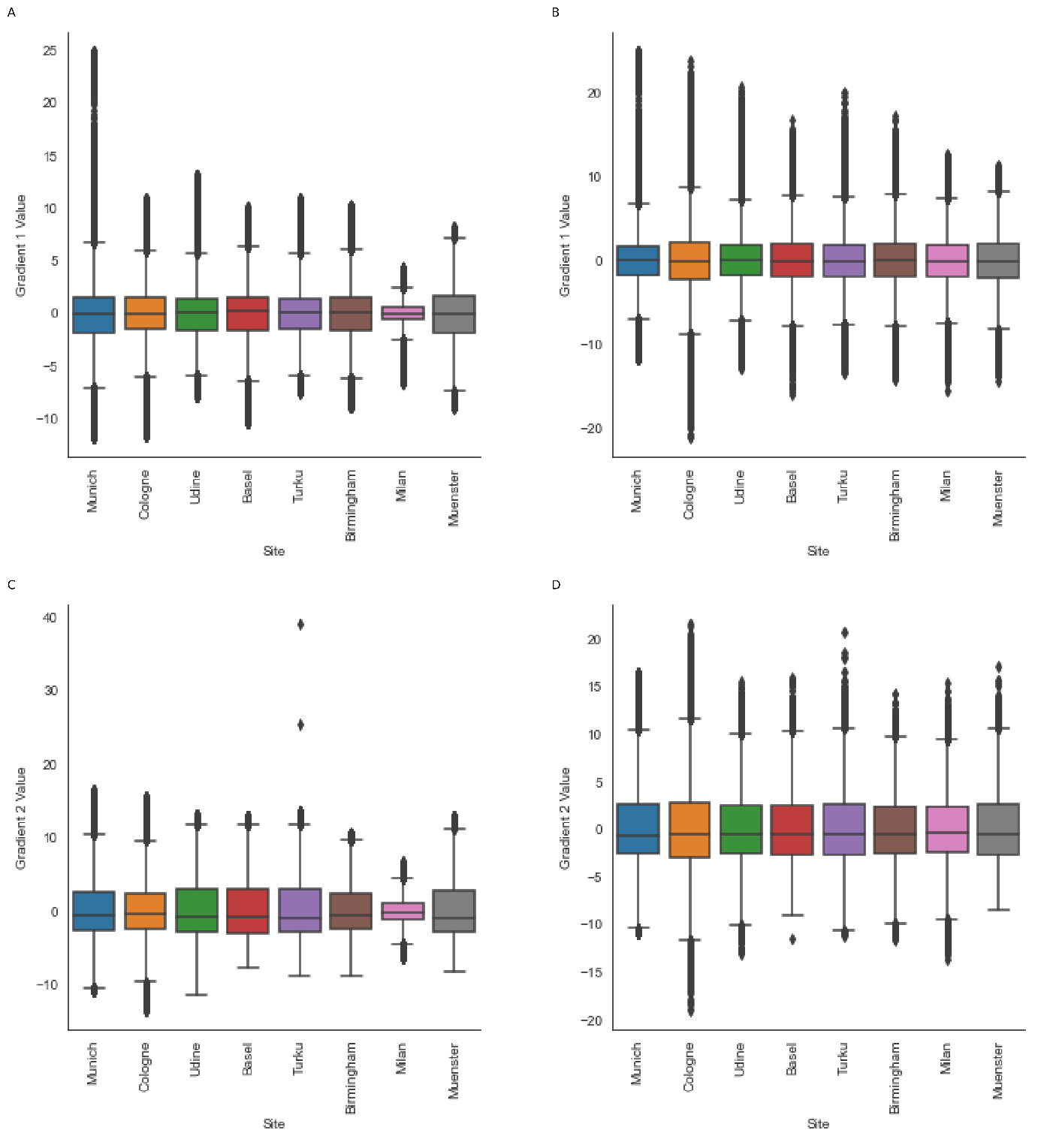
**Supplementary Figure 5.** Gradient values per site before and after COMBAT. A) Gradient 1 values before correction. B) Gradient 1 values after correction. C) Gradient 2 values before correction. D) Gradient 2 values after correction.


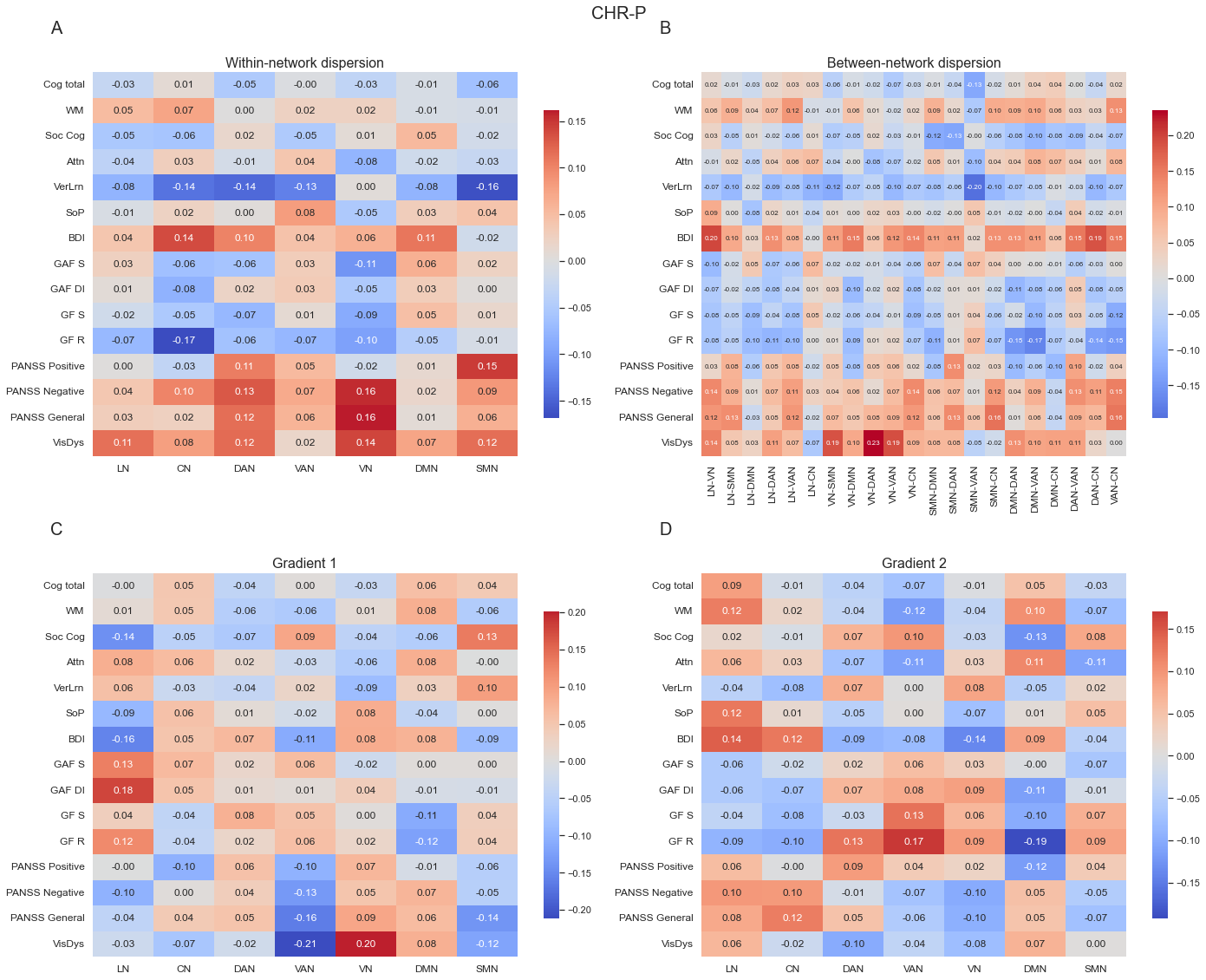


**Supplementary Figure 6.** Association of clinical variables with gradient measures for CHR-P patients. Asterisks indicate significant correlations after FDR-correction; * *p*_FDR_ < .05. (A) Correlations of within-network dispersion with clinical variables. (B) Correlations of between-network dispersion with clinical variables. (C) Correlations of mean-network values of gradient 1 with clinical variables. (D) Correlations of mean-network values of gradient 2 with clinical variables. Attn = attention; BDI = Becks Depression Inventory; CN = control network; Cog total = cognition total score; DAN = dorsal attention network; DMN = default-mode network; GAF DI = Global Assessment of Functioning Disability; GAF S = Global Assessment of Functioning Symptoms; GF R = Global Functioning Role Scale; GF S = Global Functioning Social Scale; LN = limbic network; Soc Cog = social cognition; SoP = speed of processing; SMN = somatomotor network; VAN = ventral attention network; VerLrn = verbal learning; VisDys = visual dysfunctions; VN = visual network; WM = working memory.


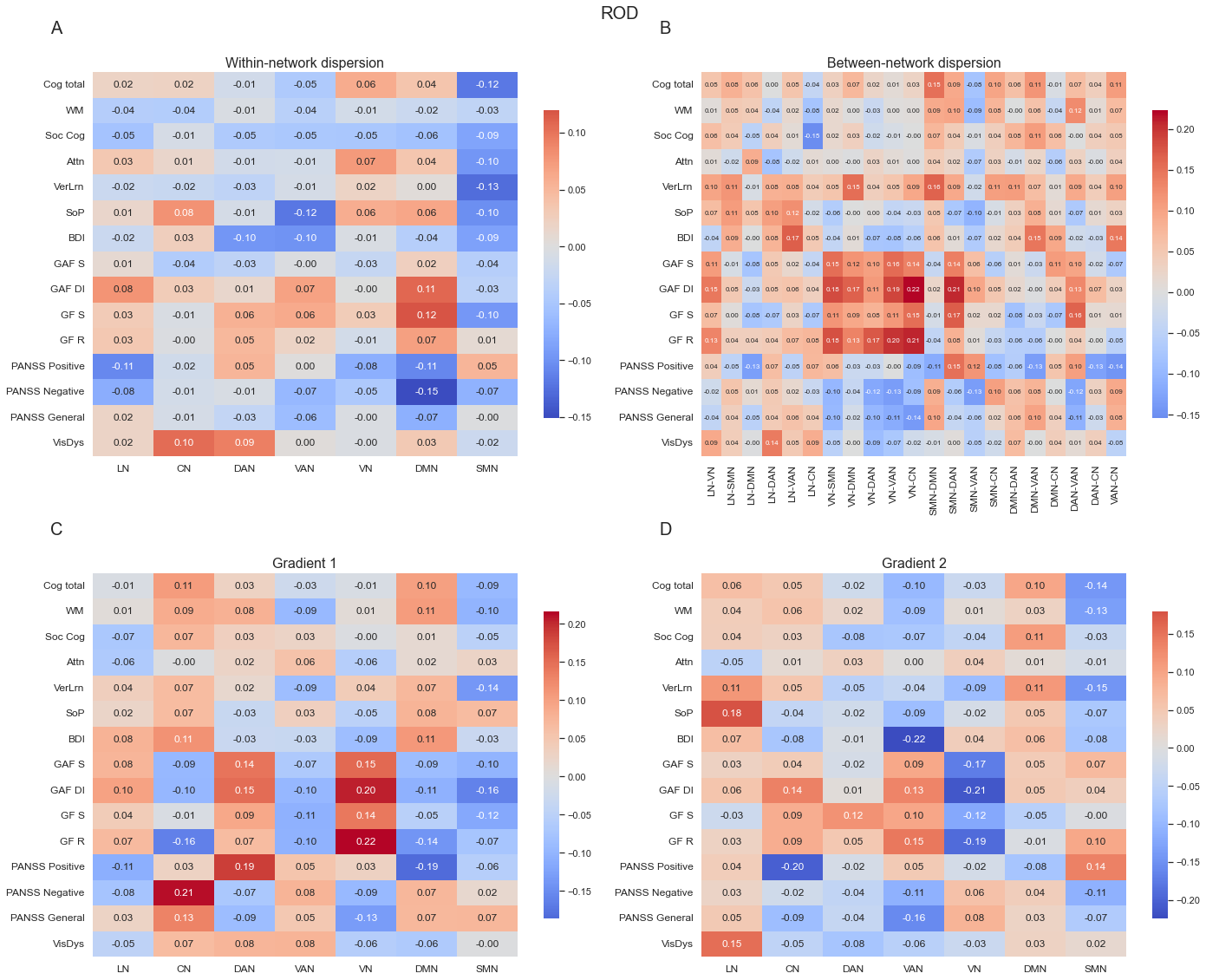


**Supplementary Figure 7.** Association of clinical variables with gradient measures for ROD patients. Asterisks indicate significant correlations after FDR-correction; * *p*_FDR_ < .05. (A) Correlations of within-network dispersion with clinical variables. (B) Correlations of between-network dispersion with clinical variables. (C) Correlations of mean-network values of gradient 1 with clinical variables. (D) Correlations of mean-network values of gradient 2 with clinical variables. Attn = attention; BDI = Becks Depression Inventory; CN = control network; Cog total = cognition total score; DAN = dorsal attention network; DMN = default-mode network; GAF DI = Global Assessment of Functioning Disability; GAF S = Global Assessment of Functioning Symptoms; GF R = Global Functioning Role Scale; GF S = Global Functioning Social Scale; LN = limbic network; Soc Cog = social cognition; SoP = speed of processing; SMN = somatomotor network; VAN = ventral attention network; VerLrn = verbal learning; VisDys = visual dysfunctions; VN = visual network; WM = working memory.


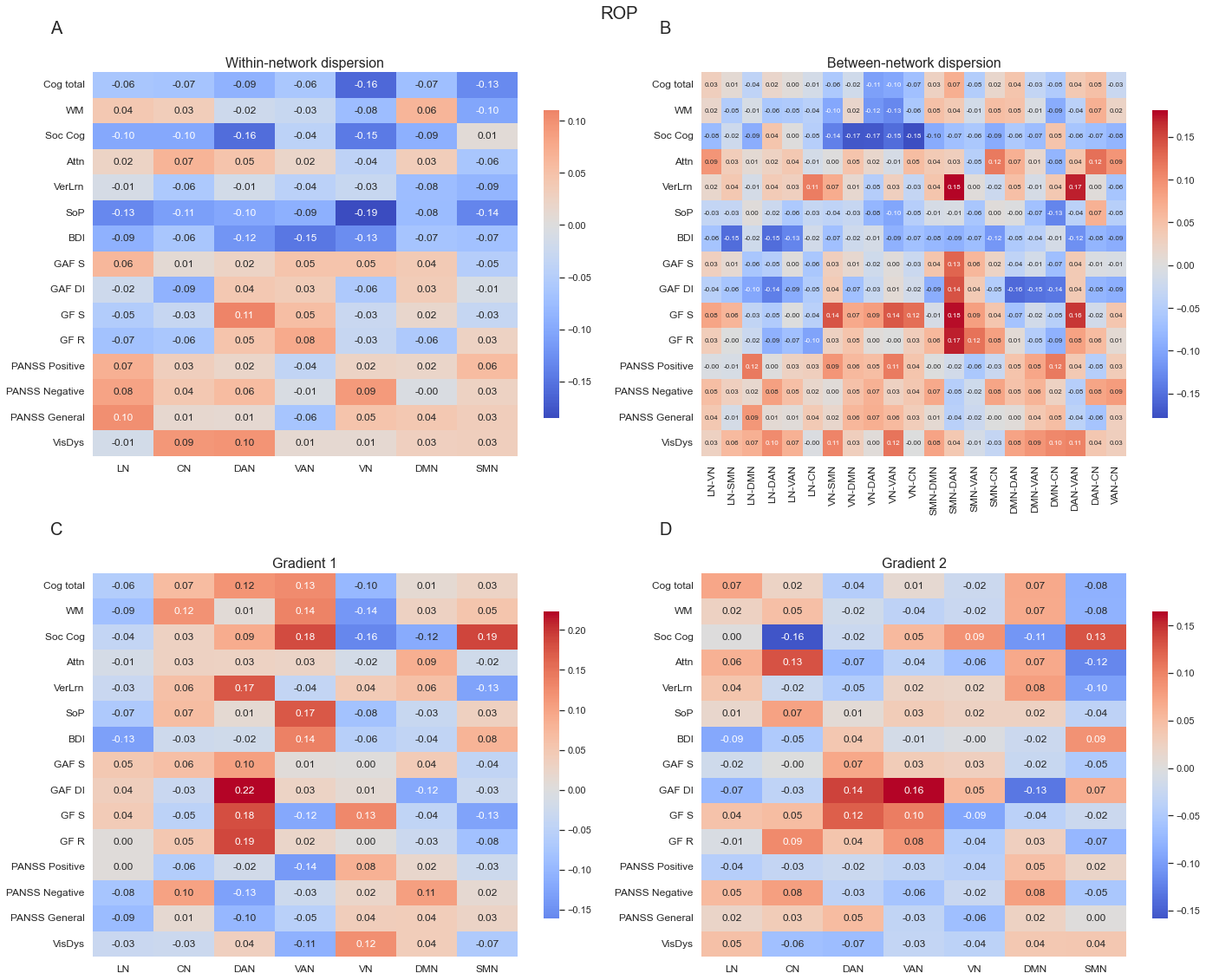


**Supplementary Figure 8.** Association of clinical variables with gradient measures for ROP patients. Asterisks indicate significant correlations after FDR-correction; * *p*_FDR_ < .05. (A) Correlations of within-network dispersion with clinical variables. (B) Correlations of between-network dispersion with clinical variables. (C) Correlations of mean-network values of gradient 1 with clinical variables. (D) Correlations of mean-network values of gradient 2 with clinical variables. Attn = attention; BDI = Becks Depression Inventory; CN = control network; Cog total = cognition total score; DAN = dorsal attention network; DMN = default-mode network; GAF DI = Global Assessment of Functioning Disability; GAF S = Global Assessment of Functioning Symptoms; GF R = Global Functioning Role Scale; GF S = Global Functioning Social Scale; LN = limbic network; Soc Cog = social cognition; SoP = speed of processing; SMN = somatomotor network; VAN = ventral attention network; VerLrn = verbal learning; VisDys = visual dysfunctions; VN = visual network; WM = working memory.


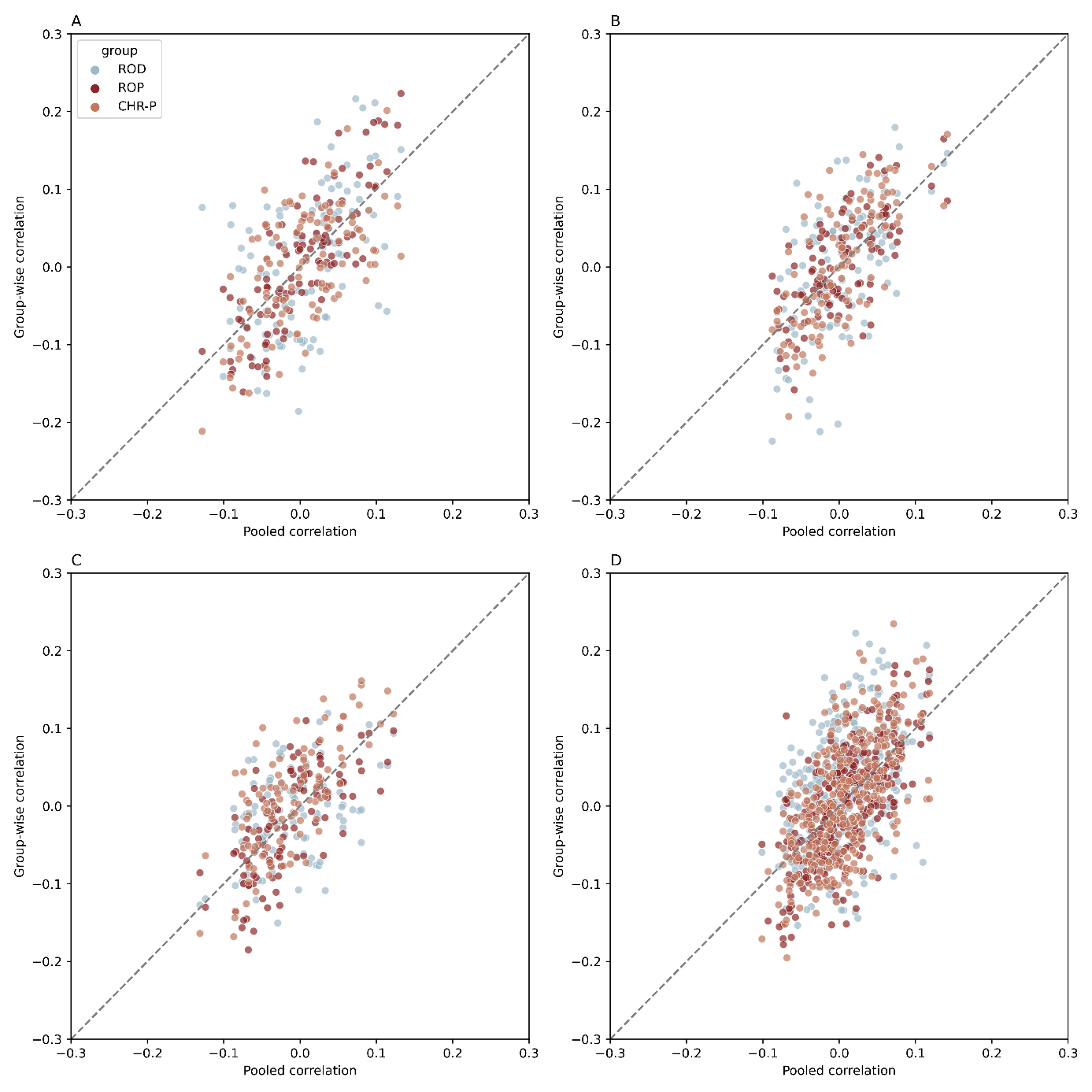


**Supplementary Figure 9.** Pooled vs. group-wise correlations of gradient variables and clinical measures. (A) Gradient 1. (B) Gradient 2. (C) Within-network dispersion. (D) Between-network dispersion. CHR-P = clinical high-risk; ROD = recent-onset depression; ROP = recent-onset psychosis.


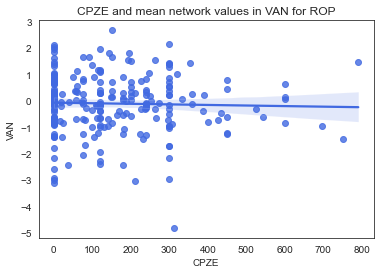


**Supplementary Figure 10.** Correlation of CPZE and mean VAN values for ROP. *r* = -.04, *p* = .581; 7 patients were excluded for this analysis because of depot medication.


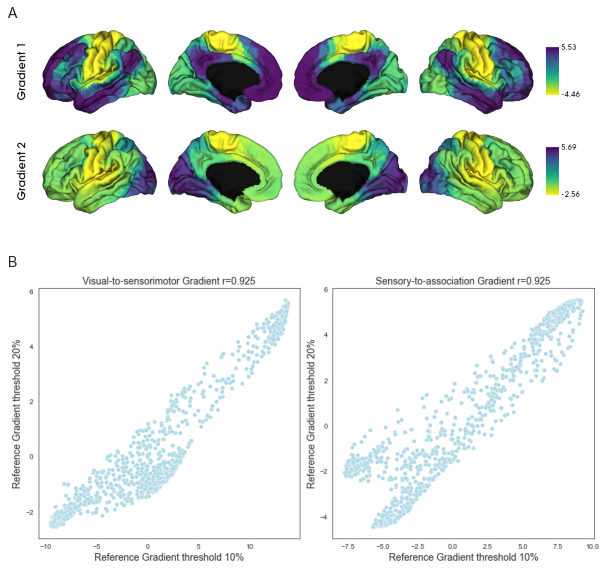


**Supplementary Figure 11.** Similarity of reference gradients with different thresholds. (A) Spatial topography of first and second gradient computed with a threshold of retaining the 20% strongest connections of the functional connectivity matrix before computing the affinity matrix. (B) Correlations of gradients with 10% and 20% thresholds. Reference gradients computed with the 20% threshold are switched in their order, but visual-to-sensorimotor gradients and sensory-to-association gradients show a high correspondence.


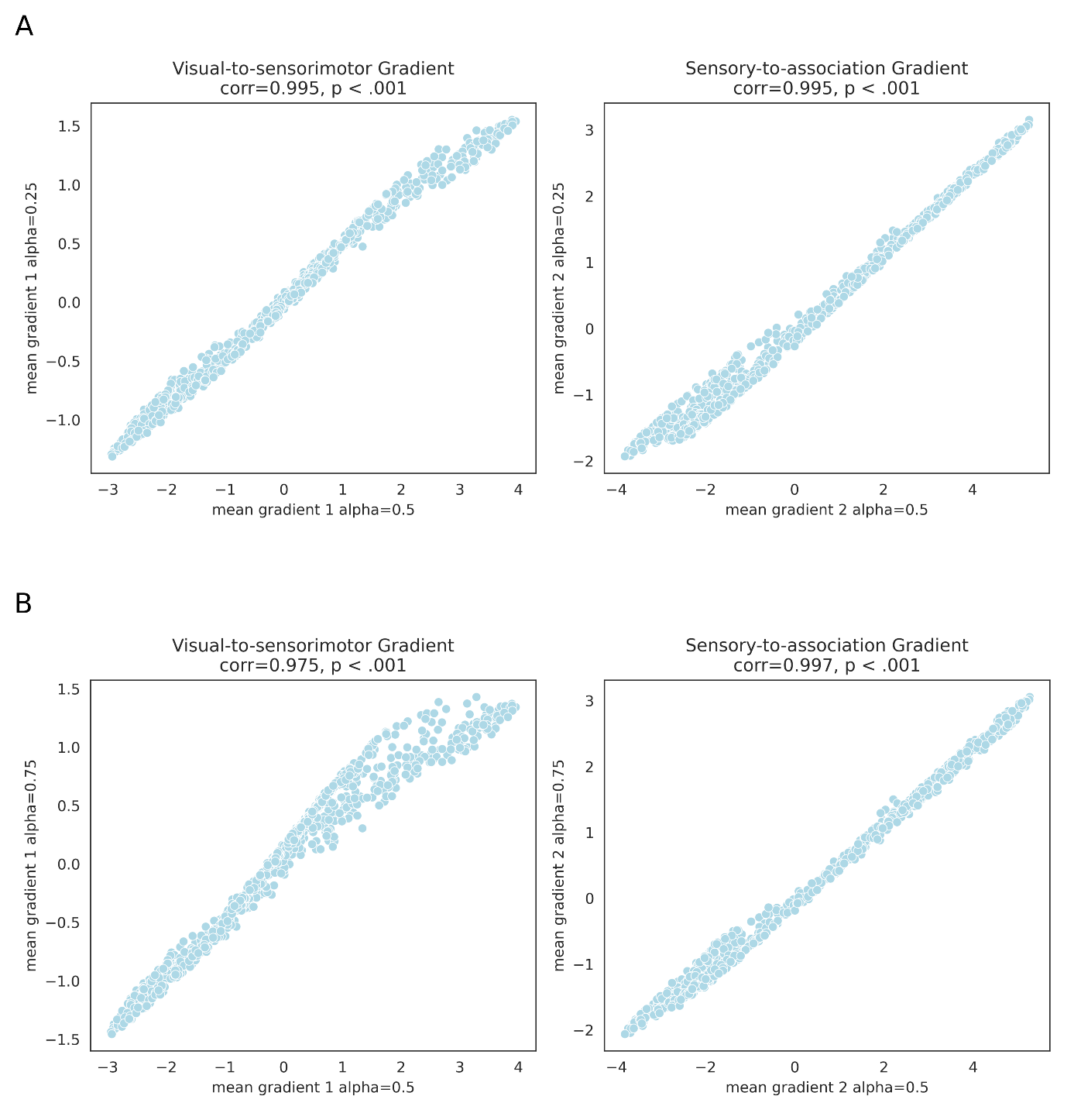


**Supplementary Figure 12.** Similarity of gradients depending on alpha parameter. (A) Correlation between mean gradients computed with alpha=0.5 and alpha=0.25. (B) Correlation between mean gradients computed with alpha=0.5 and alpha=0.75.


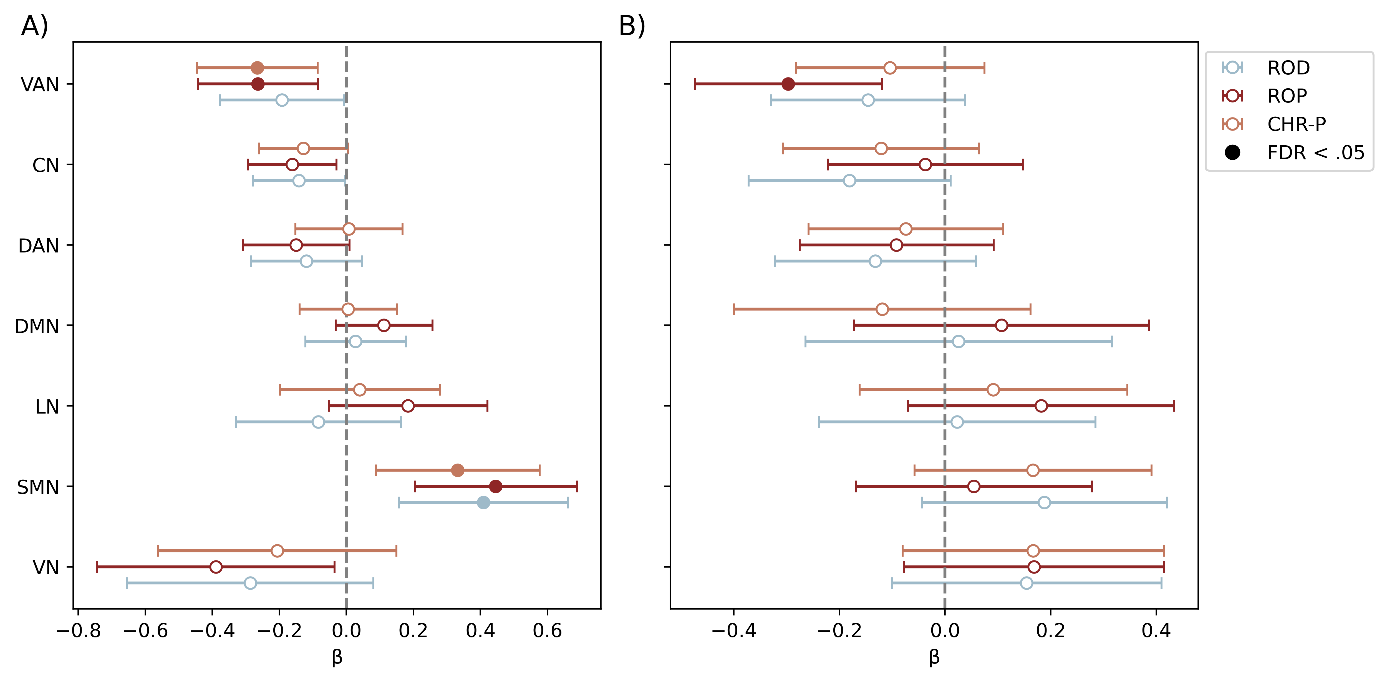


**Supplementary Figure 13.** Differences between patient groups and healthy controls in mean network values assessed by general linear models. (A) Gradient 1 for HC vs. clinical groups. (B) Gradient 2 for HC vs. clinical groups. CHR-P = clinical high-risk; CN = control network; DAN = dorsal attention network; DMN = default mode network; HC = healthy controls; LN = limbic network; ROD = recent-onset depression; ROP = recent-onset psychosis; SMN = somatomotor network; VAN = ventral attention network; VN = visual network.


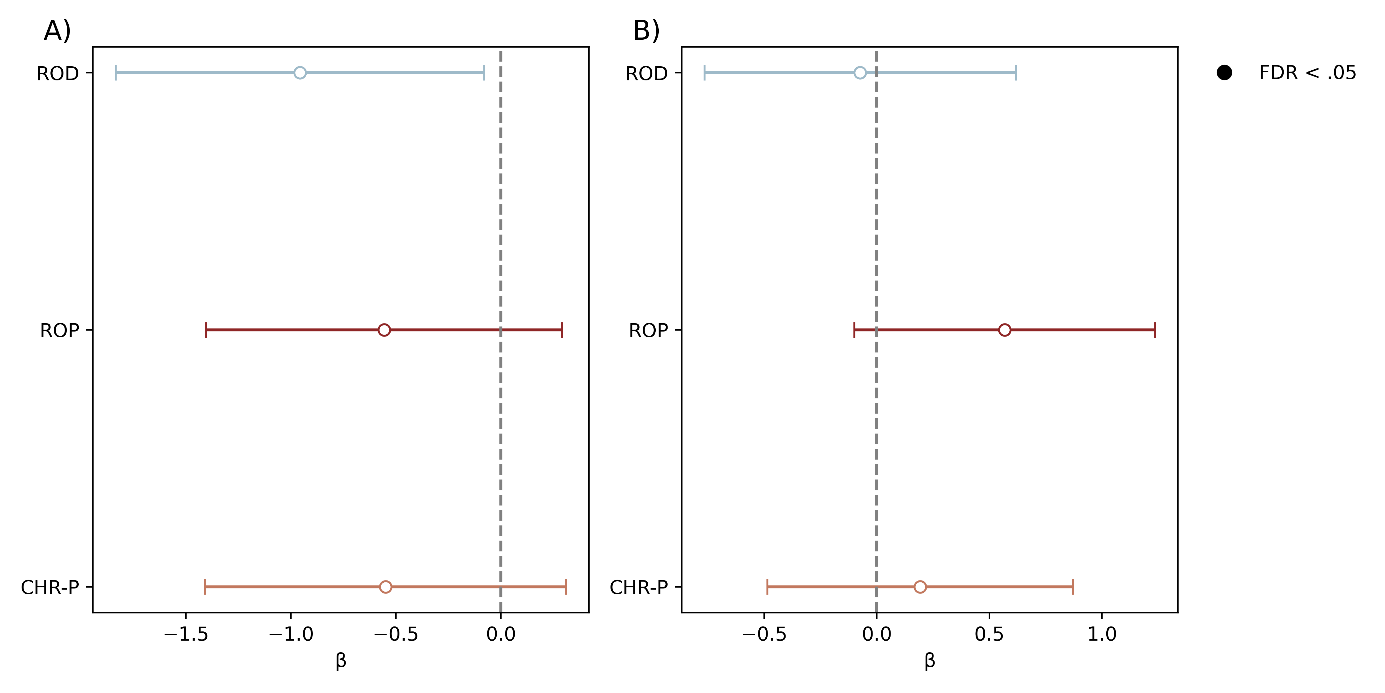


**Supplementary Figure 14.** Differences between patient groups and healthy controls in gradient assessed by general linear models. (A) Gradient 1 for HC vs. clinical groups. (B) Gradient 2 for HC vs. clinical groups. CHR-P = clinical high-risk; CN = control network; DAN = dorsal attention network; DMN = default mode network; HC = healthy controls; LN = limbic network; ROD = recent-onset depression; ROP = recent-onset psychosis; SMN = somatomotor network; VAN = ventral attention network; VN = visual network.


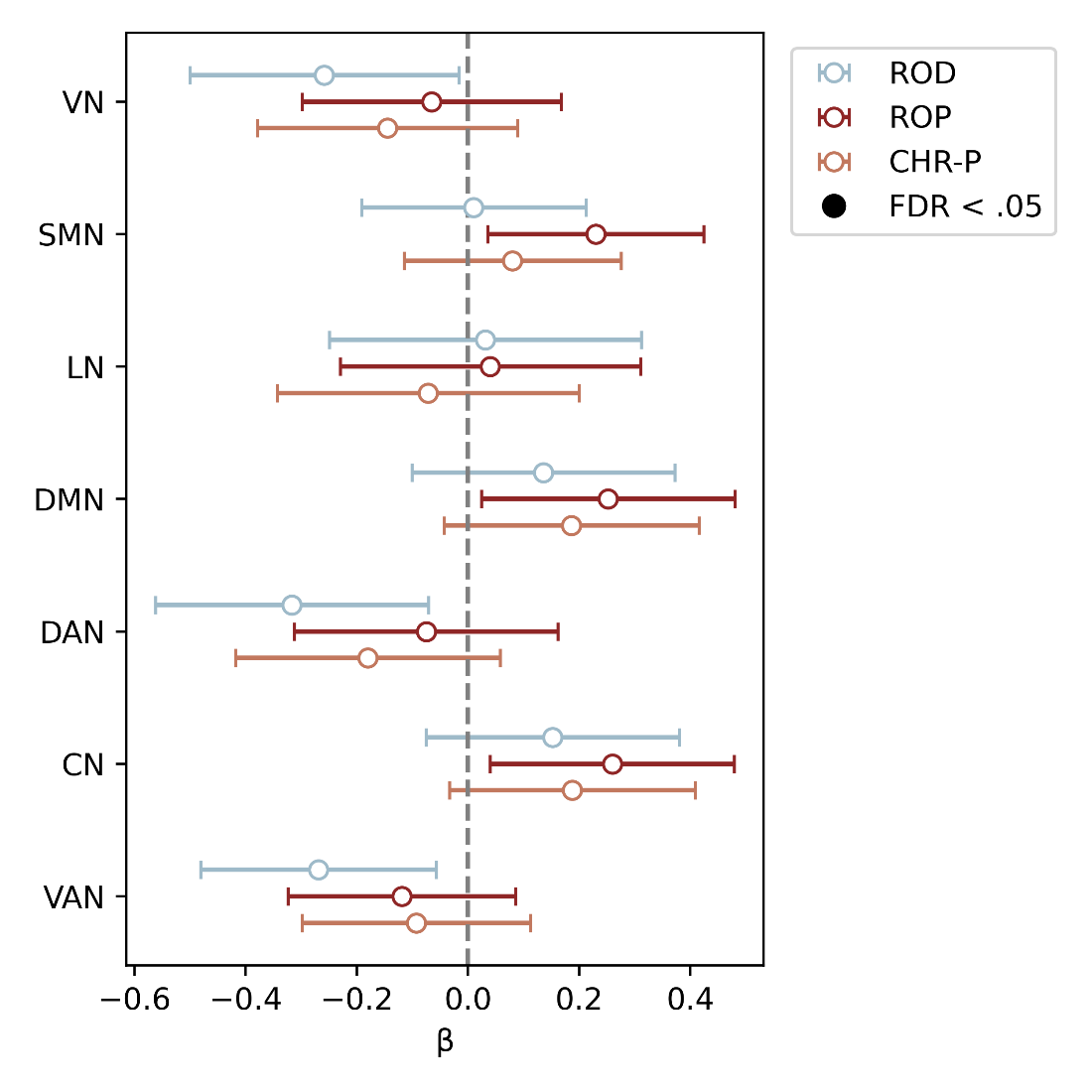


**Supplementary Figure 15.** Differences between patient groups and healthy controls in within-network dispersion assessed by general linear models. CHR-P = clinical high-risk; CN = control network; DAN = dorsal attention network; DMN = default mode network; HC = healthy controls; LN = limbic network; ROD = recent-onset depression; ROP = recent-onset psychosis; SMN = somatomotor network; VAN = ventral attention network; VN = visual network.


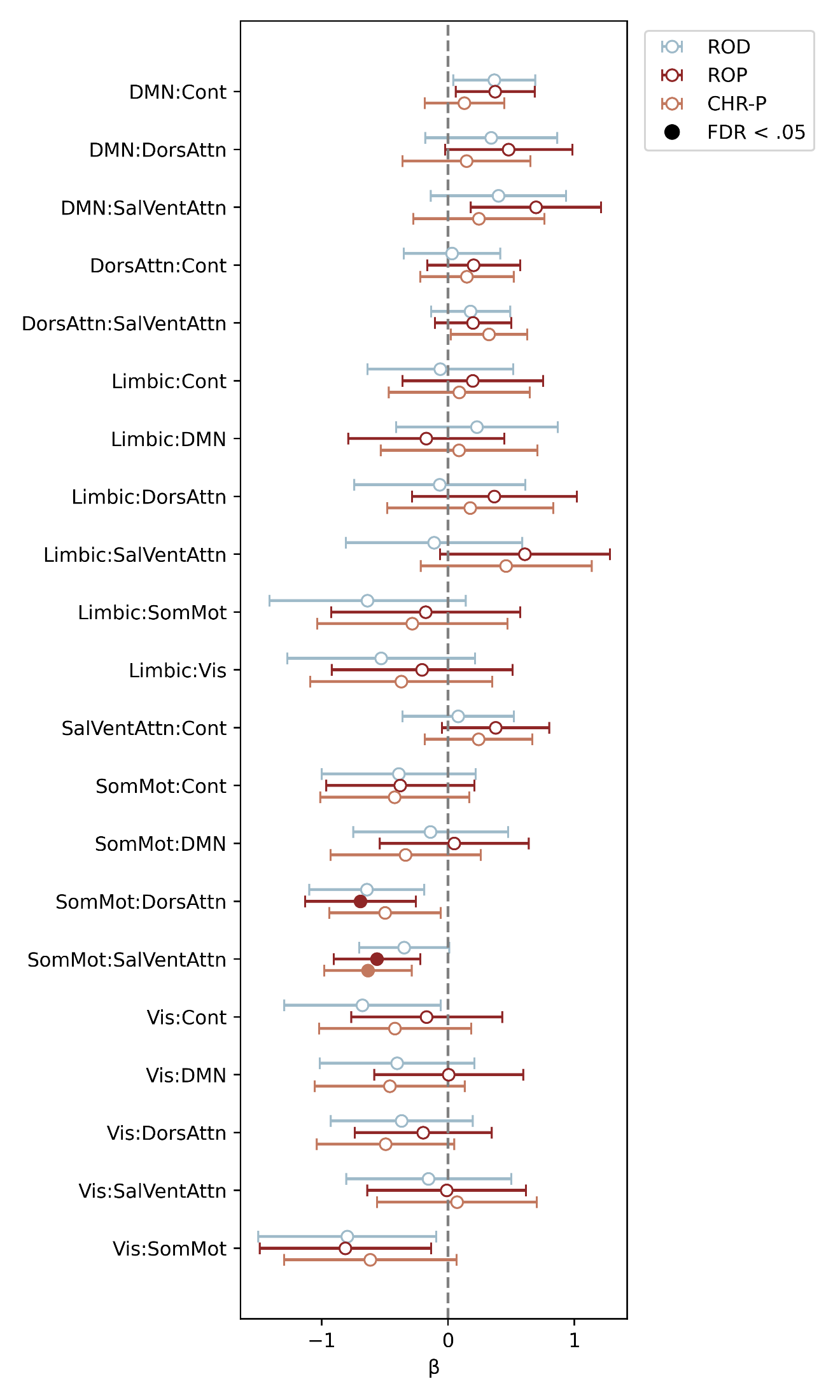


**Supplementary Figure 16.** Differences between patient groups and healthy controls in between-network dispersion assessed by general linear models. CHR-P = clinical high-risk; CN = control network; DAN = dorsal attention network; DMN = default mode network; HC = healthy controls; LN = limbic network; ROD = recent-onset depression; ROP = recent-onset psychosis; SMN = somatomotor network; VAN = ventral attention network; VN = visual network.


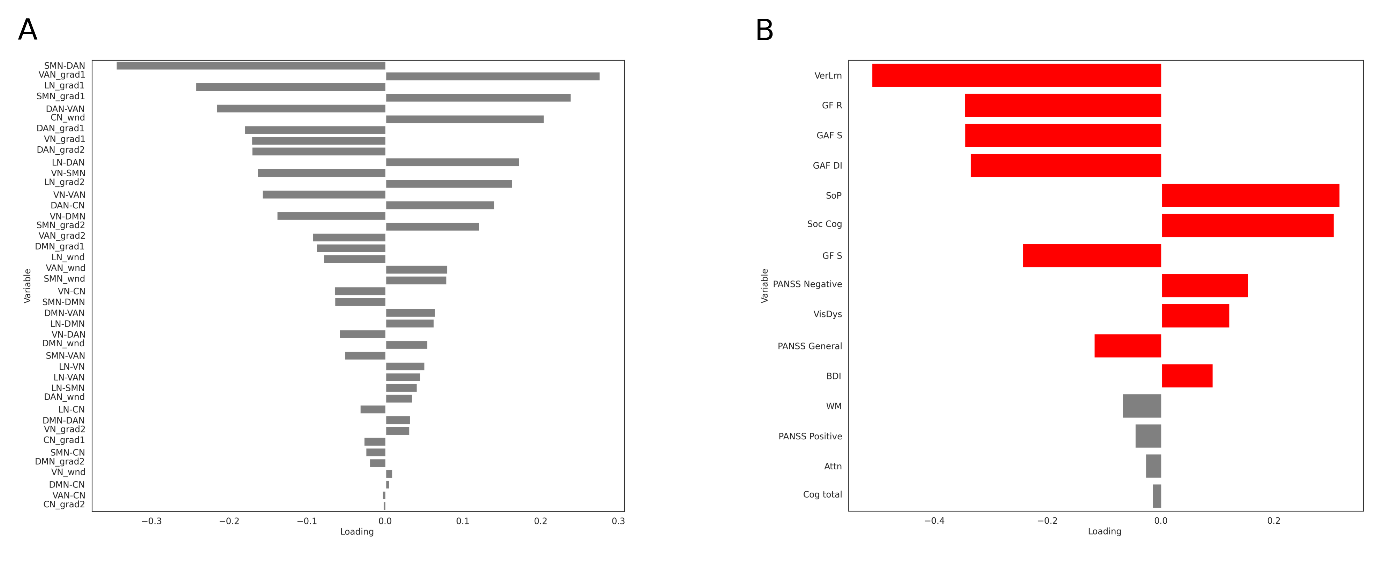


**Supplementary Figure 17.** Loadings of variable sets on first canonical component. (A) Loadings of gradient variables on component. (B) Loadings of clinical variables on component. Color indicates significant loadings after FDR-correction. Attn = attention; BDI = Becks Depression Inventory; CN = control network; Cog total = cognition total score; DAN = dorsal attention network; DMN = default-mode network; GAF DI = Global Assessment of Functioning Disability; GAF S = Global Assessment of Functioning Symptoms; GF R = Global Functioning Role Scale; GF S = Global Functioning Social Scale; LN = limbic network; Soc Cog = social cognition; SoP = speed of processing; SMN = somatomotor network; VAN = ventral attention network; VerLrn = verbal learning; VisDys = visual dysfunctions; VN = visual network; WM = working memory.

**The PRONIA CONSORTIUM**

Nikolaos Koutsouleris, Anne Ruef, Lisa Hahn, Dominic B. Dwyer, Shalaila Haas, Linda A. Antonucci, Alkomiet Hasan, Claudius Hoff, Ifrah Khanyaree, Aylin Melo, Susanna Muckenhuber-Sternbauer, Yanis Köhler, Ömer Öztürk, Nora Penzel, David Popovic, Adrian Rangnick, Sebastian von Saldern, Rachele Sanfelici, Moritz Spangemacher, Ana Tupac, Maria Fernanda Urquijo-Castro, Johanna Weiske, Antonia Wosgien, Camilla Krämer, Lana Kambeitz-Ilankovic, Joseph Kambeitz, Julian Wenzel, Stephan Ruhrmann, Karsten Blume, Dennis Hedderich, Dominika Julkowski, Nathalie Kaiser, Thorsten Lichtenstein, Ruth Milz, Alexandra Nikolaides, Tanja Pilgram, Mauro Seves, Martina Wassen, Christina Andreou, Stefan Borgwardt, André Schmidt, Anita Riecher-Rössler, Laura Egloff, Fabienne Harrisberger, Ulrike Heitz, Claudia Lenz, Letizia Leanza, Amatya Mackintosh, Renata Smieskova, Erich Studerus, Anna Walter, Sonja Widmayer, Stephen J. Wood, Rachel Upthegrove, Paris Alexandros Lalousis, Chris Day, Sian Lowri Griffiths, Mariam Iqbal, Mirabel Pelton, Pavan Mallikarjun, Alexandra Stainton, Ashleigh Lin, Jarmo Hietala, Raimo K. R. Salokangas, Alexander Denissoff, Anu Ellilä, Tiina From, Markus Heinimaa, Tuula Ilonen, Päivi Jalo, Heikki Laurikainen, Antti Luutonen, Akseli Mäkela, Janina Paju, Henri Pesonen, Reetta-Liina Säilä, Anna Toivonen, Otto Turtonen, Frauke Schultze-Lutter, Eva Meisenzahl, Alexandra Korda, Susanne Neufang, Christian Schmidt-Kraepelin, Henrik Rohner, Sonja Botterweck, Norman Kluthausen, Gerald Antoch, Julian Caspers, Hans-Jörg Wittsack, Pierluigi Selvaggi, Alessandro Bertolino, Giuseppe Blasi, Giulio Pergola, Grazia Caforio, Leonardo Fazio, Tiziana Quarto, Barbara Gelao, Raffaella Romano, Ileana Andriola, Andrea Falsetti, Marina Barone, Roberta Passiatore, Marina Sangiuliano, Rebekka Lencer, Marian Surmann, Olga Bienek, Udo Dannlowski, Ana Beatriz Solana, Manuela Abraham,Timo Schirmer, Paolo Brambilla, Carlo Altamura, Marika Belleri, Francesca Bottinelli, Adele Ferro, Marta Re, Emiliano Monzani, Maurizio Sberna, Giampaolo Perna, Maria Nobile, Alessandra Alciati, Armando D’Agostino, Lorenzo Del Fabro, Matteo Balestrieri, Carolina Bonivento, Giuseppe Cabras, Franco Fabbro, Marco Garzitto, Sara Piccin, Christos Pantelis and Christos Davatzikos

**References**

1. First MB, Gibbon M. The Structured Clinical Interview for DSM-IV Axis I Disorders (SCID-I) and the Structured Clinical Interview for DSM-IV Axis II Disorders (SCID-II). In: Comprehensive handbook of psychological assessment, Vol. 2: Personality assessment. Hoboken, NJ, US: John Wiley & Sons, Inc.; 2004. p. 134–43.

2. http://www.dgppn.de/fileadmin/user_upload/_medien/download/pdf/kurzversion-leitlinien/s3-praxisleitlinienbd1-schizophrenie.pdf [Internet]. [cited 2013 Dec 4]. Available from: http://www.dgppn.de/fileadmin/user_upload/_medien/download/pdf/kurzversion-leitlinien/s3-praxisleitlinienbd1-schizophrenie.pdf

3. McGlashan TH, Miller TJ, Woods SW, Hoffman RE, Davidson L. Instrument for the Assessment of Prodromal Symptoms and States. In: Miller T, Mednick SA, McGlashan TH, Libiger J, Johannessen JO, editors. Early Intervention in Psychotic Disorders [Internet]. Dordrecht: Springer Netherlands; 2001 [cited 2024 Apr 4]. p. 135–49. Available from: http://link.springer.com/10.1007/978-94-010-0892-1_7 doi:10.1007/978-94-010-0892-1_7

4. Schultze-Lutter F, Addington J, Ruhrmann S, Klosterkötter J. Schizophrenia proneness instrument, adult version (SPI-A). Rome Giovanni Fioriti. 2007.

5. Kern RS, Nuechterlein KH, Green MF, Baade LE, Fenton WS, Gold JM, et al. The MATRICS Consensus Cognitive Battery, Part 2: Co-Norming and Standardization. Am J Psychiatry. 2008 Feb;165(2):214–20. doi:10.1176/appi.ajp.2007.07010043

6. Nuechterlein KH, Green MF, Kern RS, Baade LE, Barch DM, Cohen JD, et al. The MATRICS Consensus Cognitive Battery, Part 1: Test Selection, Reliability, and Validity. Am J Psychiatry. 2008 Feb;165(2):203–13. doi:10.1176/appi.ajp.2007.07010042

7. Nowicki S, Duke MP. Individual differences in the nonverbal communication of affect: The diagnostic analysis of nonverbal accuracy scale. J Nonverbal Behav. 1994 Mar;18(1):9–35. doi:10.1007/BF02169077

8. Strauss E, Sherman EMS, Spreen O, Spreen O. A compendium of neuropsychological tests: administration, norms, and commentary. 3rd ed. Oxford ; New York: Oxford University Press; 2006. 1216 p.

9. Lezak MD. Neuropsychological assessment. Oxford University Press, USA; 2004.

10. Tombaugh T. Trail Making Test A and B: Normative data stratified by age and education. Arch Clin Neuropsychol. 2004 Mar;19(2):203–14. doi:10.1016/S0887-6177(03)00039-8

11. Cornblatt BA, Risch NJ, Faris G, Friedman D, Erlenmeyer-Kimling L. The continuous performance test, identical pairs version (CPT-IP): I. new findings about sustained attention in normal families. Psychiatry Res. 1988 Nov;26(2):223–38. doi:10.1016/0165-1781(88)90076-5

12. Wechsler D. Manual for the Wechsler Adult Intelligence Scale. Oxford, England: Psychological Corp.; 1955. vi, 110 p. (Manual for the Wechsler Adult Intelligence Scale.).

13. Ruff R. Benton controlled oral word association test: Reliability and updated norms. Arch Clin Neuropsychol. 1996;11(4):329–38. doi:10.1016/0887-6177(95)00033-X

14. Eliason MJ, Richman LC. The Continuous Performance Test in Learning Disabled and Nondisabled Children. J Learn Disabil. 1987 Dec;20(10):614–9. doi:10.1177/002221948702001007

15. Schmidt M. Rey auditory verbal learning test. Western Psychological Services Los Angeles; 1996.

16. Wenzel J, Haas SS, Dwyer DB, Ruef A, Oeztuerk OF, Antonucci LA, et al. Cognitive subtypes in recent onset psychosis: distinct neurobiological fingerprints? Neuropsychopharmacology. 2021 Jul;46(8):1475–83. doi:10.1038/s41386-021-00963-1

17. Bayer JMM, Thompson PM, Ching CRK, Liu M, Chen A, Panzenhagen AC, et al. Site effects how-to and when: An overview of retrospective techniques to accommodate site effects in multi-site neuroimaging analyses. Front Neurol. 2022 Oct 31;13:923988. doi:10.3389/fneur.2022.923988

18. Pomponio R, Erus G, Habes M, Doshi J, Srinivasan D, Mamourian E, et al. Harmonization of large MRI datasets for the analysis of brain imaging patterns throughout the lifespan. NeuroImage. 2020 Mar;208:116450. doi:10.1016/j.neuroimage.2019.116450

19. Wang HT, Smallwood J, Mourao-Miranda J, Xia CH, Satterthwaite TD, Bassett DS, et al. Finding the needle in a high-dimensional haystack: Canonical correlation analysis for neuroscientists. NeuroImage. 2020 Aug;216:116745. doi:10.1016/j.neuroimage.2020.116745

20. Buciuman MO, Oeztuerk OF, Popovic D, Enrico P, Ruef A, Bieler N, et al. Structural and Functional Brain Patterns Predict Formal Thought Disorder’s Severity and Its Persistence in Recent-Onset Psychosis: Results From the PRONIA Study. Biol Psychiatry Cogn Neurosci Neuroimaging. 2023 Dec;8(12):1207–17. doi:10.1016/j.bpsc.2023.06.001

21. Aghourian M, Legault-Denis C, Soucy JP, Rosa-Neto P, Gauthier S, Kostikov A, et al. Quantification of brain cholinergic denervation in Alzheimer’s disease using PET imaging with [18F]-FEOBV. Mol Psychiatry. 2017 Nov;22(11):1531–8. doi:10.1038/mp.2017.183

22. Bedard MA, Aghourian M, Legault-Denis C, Postuma RB, Soucy JP, Gagnon JF, et al. Brain cholinergic alterations in idiopathic REM sleep behaviour disorder: a PET imaging study with 18F-FEOBV. Sleep Med. 2019 Jun;58:35–41. doi:10.1016/j.sleep.2018.12.020

23. Hansen JY, Shafiei G, Markello RD, Smart K, Cox SML, Nørgaard M, et al. Mapping neurotransmitter systems to the structural and functional organization of the human neocortex. Nat Neurosci. 2022 Nov;25(11):1569–81. doi:10.1038/s41593-022-01186-3

24. Naganawa M, Nabulsi N, Henry S, Matuskey D, Lin SF, Slieker L, et al. First-in-Human Assessment of^11^ C-LSN3172176, an M1 Muscarinic Acetylcholine Receptor PET Radiotracer. J Nucl Med. 2021 Apr;62(4):553–60. doi:10.2967/jnumed.120.246967

25. Hillmer AT, Esterlis I, Gallezot JD, Bois F, Zheng MQ, Nabulsi N, et al. Imaging of cerebral α4β2* nicotinic acetylcholine receptors with (−)-[18F]Flubatine PET: Implementation of bolus plus constant infusion and sensitivity to acetylcholine in human brain. NeuroImage. 2016 Nov;141:71–80. doi:10.1016/j.neuroimage.2016.07.026

26. Malén T, Karjalainen T, Isojärvi J, Vehtari A, Bürkner PC, Putkinen V, et al. Atlas of type 2 dopamine receptors in the human brain: Age and sex dependent variability in a large PET cohort. NeuroImage. 2022 Jul;255:119149. doi:10.1016/j.neuroimage.2022.119149

27. Sandiego CM, Gallezot JD, Lim K, Ropchan J, Lin S fei, Gao H, et al. Reference Region Modeling Approaches for Amphetamine Challenge Studies with [^11^ C]FLB 457 and PET. J Cereb Blood Flow Metab. 2015 Apr;35(4):623–9. doi:10.1038/jcbfm.2014.237

28. Smith CT, Crawford JL, Dang LC, Seaman KL, San Juan MD, Vijay A, et al. Partial-volume correction increases estimated dopamine D2-like receptor binding potential and reduces adult age differences. J Cereb Blood Flow Metab. 2019 May;39(5):822–33. doi:10.1177/0271678X17737693

29. Jaworska N, Cox SML, Tippler M, Castellanos-Ryan N, Benkelfat C, Parent S, et al. Extra-striatal D2/3 receptor availability in youth at risk for addiction. Neuropsychopharmacology. 2020 Aug;45(9):1498–505. doi:10.1038/s41386-020-0662-7

30. Alakurtti K, Johansson JJ, Joutsa J, Laine M, Bäckman L, Nyberg L, et al. Long-Term Test–Retest Reliability of Striatal and Extrastriatal Dopamine D_2/3_ Receptor Binding: Study with [^11^ C]Raclopride and High-Resolution PET. J Cereb Blood Flow Metab. 2015 Jul;35(7):1199–205. doi:10.1038/jcbfm.2015.53

31. Dukart J, Holiga Š, Chatham C, Hawkins P, Forsyth A, McMillan R, et al. Cerebral blood flow predicts differential neurotransmitter activity. Sci Rep. 2018 Mar 6;8(1):4074. doi:10.1038/s41598-018-22444-0

32. Sasaki T, Ito H, Kimura Y, Arakawa R, Takano H, Seki C, et al. Quantification of Dopamine Transporter in Human Brain Using PET with^18^ F-FE-PE2I. J Nucl Med. 2012 Jul;53(7):1065–73. doi:10.2967/jnumed.111.101626

33. Kaller S, Rullmann M, Patt M, Becker GA, Luthardt J, Girbardt J, et al. Test–retest measurements of dopamine D1-type receptors using simultaneous PET/MRI imaging. Eur J Nucl Med Mol Imaging. 2017 Jun;44(6):1025–32. doi:10.1007/s00259-017-3645-0

34. Laurikainen H, Tuominen L, Tikka M, Merisaari H, Armio RL, Sormunen E, et al. Sex difference in brain CB1 receptor availability in man. NeuroImage. 2019 Jan;184:834–42. doi:10.1016/j.neuroimage.2018.10.013

35. Normandin MD, Zheng MQ, Lin KS, Mason NS, Lin SF, Ropchan J, et al. Imaging the Cannabinoid CB1 Receptor in Humans with [^11^ C] OMAR: Assessment of Kinetic Analysis Methods, Test–Retest Reproducibility, and Gender Differences. J Cereb Blood Flow Metab. 2015 Aug;35(8):1313–22. doi:10.1038/jcbfm.2015.46

36. Vijay A, Cavallo D, Goldberg A, De Laat B, Nabulsi N, Huang Y, et al. PET imaging reveals lower kappa opioid receptor availability in alcoholics but no effect of age. Neuropsychopharmacology. 2018 Dec;43(13):2539–47. doi:10.1038/s41386-018-0199-1

37. Kantonen T, Karjalainen T, Isojärvi J, Nuutila P, Tuisku J, Rinne J, et al. Interindividual variability and lateralization of μ-opioid receptors in the human brain. NeuroImage. 2020 Aug;217:116922. doi:10.1016/j.neuroimage.2020.116922

38. Turtonen O, Saarinen A, Nummenmaa L, Tuominen L, Tikka M, Armio RL, et al. Adult Attachment System Links With Brain Mu Opioid Receptor Availability In Vivo. Biol Psychiatry Cogn Neurosci Neuroimaging. 2021 Mar;6(3):360–9. doi:10.1016/j.bpsc.2020.10.013

39. DuBois JM, Rousset OG, Rowley J, Porras-Betancourt M, Reader AJ, Labbe A, et al. Characterization of age/sex and the regional distribution of mGluR5 availability in the healthy human brain measured by high-resolution [11C]ABP688 PET. Eur J Nucl Med Mol Imaging. 2016 Jan;43(1):152–62. doi:10.1007/s00259-015-3167-6

40. Smart K, Cox SML, Scala SG, Tippler M, Jaworska N, Boivin M, et al. Sex differences in [11C]ABP688 binding: a positron emission tomography study of mGlu5 receptors. Eur J Nucl Med Mol Imaging. 2019 May;46(5):1179–83. doi:10.1007/s00259-018-4252-4

41. Savli M, Bauer A, Mitterhauser M, Ding YS, Hahn A, Kroll T, et al. Normative database of the serotonergic system in healthy subjects using multi-tracer PET. NeuroImage. 2012 Oct;63(1):447–59. doi:10.1016/j.neuroimage.2012.07.001

42. Beliveau V, Ganz M, Feng L, Ozenne B, Højgaard L, Fisher PM, et al. A High-Resolution *In Vivo* Atlas of the Human Brain’s Serotonin System. J Neurosci. 2017 Jan 4;37(1):120–8. doi:10.1523/JNEUROSCI.2830-16.2016

43. Gallezot JD, Nabulsi N, Neumeister A, Planeta-Wilson B, Williams WA, Singhal T, et al. Kinetic Modeling of the Serotonin 5-HT_1B_ Receptor Radioligand [^11^ C]P943 in Humans. J Cereb Blood Flow Metab. 2010 Jan;30(1):196–210. doi:10.1038/jcbfm.2009.195

44. Fazio P, Schain M, Varnäs K, Halldin C, Farde L, Varrone A. Mapping the distribution of serotonin transporter in the human brainstem with high-resolution PET: Validation using postmortem autoradiography data. NeuroImage. 2016 Jun;133:313–20. doi:10.1016/j.neuroimage.2016.03.019

45. Radhakrishnan R, Nabulsi N, Gaiser E, Gallezot JD, Henry S, Planeta B, et al. Age-Related Change in 5-HT_6_ Receptor Availability in Healthy Male Volunteers Measured with^11^ C-GSK215083 PET. J Nucl Med. 2018 Sep;59(9):1445–50. doi:10.2967/jnumed.117.206516

46. Nørgaard M, Beliveau V, Ganz M, Svarer C, Pinborg LH, Keller SH, et al. A high-resolution in vivo atlas of the human brain’s benzodiazepine binding site of GABAA receptors. NeuroImage. 2021 May;232:117878. doi:10.1016/j.neuroimage.2021.117878

47. Lukow PB, Martins D, Veronese M, Vernon AC, McGuire P, Turkheimer FE, et al. Cellular and molecular signatures of in vivo imaging measures of GABAergic neurotransmission in the human brain. Commun Biol. 2022 Apr 19;5(1):372. doi:10.1038/s42003-022-03268-1

48. Hesse S, Becker GA, Rullmann M, Bresch A, Luthardt J, Hankir MK, et al. Central noradrenaline transporter availability in highly obese, non-depressed individuals. Eur J Nucl Med Mol Imaging. 2017 Jun;44(6):1056–64. doi:10.1007/s00259-016-3590-3

49. Ding YS, Singhal T, Planeta-Wilson B, Gallezot JD, Nabulsi N, Labaree D, et al. PET imaging of the effects of age and cocaine on the norepinephrine transporter in the human brain using (S,S)-[^11^ C]O-methylreboxetine and HRRT. Synapse. 2010 Jan;64(1):30–8. doi:10.1002/syn.20696

50. Gallezot JD, Planeta B, Nabulsi N, Palumbo D, Li X, Liu J, et al. Determination of receptor occupancy in the presence of mass dose: [^11^ C]GSK189254 PET imaging of histamine H_3_ receptor occupancy by PF-03654746. J Cereb Blood Flow Metab. 2017 Mar;37(3):1095–107. doi:10.1177/0271678X16650697
